# Study protocol title: a prospective cohort study of low back pain

**DOI:** 10.1186/1471-2474-14-84

**Published:** 2013-03-07

**Authors:** Arun Garg, Kurt T Hegmann, J Steven Moore, Jay Kapellusch, Matthew S Thiese, Sruthi Boda, Parag Bhoyr, Donald Bloswick, Andrew Merryweather, Richard Sesek, Gwen Deckow-Schaefer, James Foster, Eric Wood, Xiaoming Sheng, Richard Holubkov

**Affiliations:** 1 Center for Ergonomics, University of Wisconsin-Milwaukee, P.O. Box 784, Milwaukee, WI 53201, USA; 2 Rocky Mountain Center for Occupational & Environment Health, Department of Family and Preventive Medicine, University of Utah, 391 Chipeta Way, Suite C, Salt Lake City, UT 84108, USA; 3 School of Rural Public Health, Texas A&M University Health Science Center, College Station, TX 77843-1266, USA

**Keywords:** Epidemiology, Ergonomics, Cohort, Low back pain, NIOSH lifting equation

## Abstract

**Background:**

Few prospective cohort studies of workplace low back pain (LBP) with quantified job physical exposure have been performed. There are few prospective epidemiological studies for LBP occupational risk factors and reported data generally have few adjustments for many personal and psychosocial factors.

**Methods/design:**

A multi-center prospective cohort study has been incepted to quantify risk factors for LBP and potentially develop improved methods for designing and analyzing jobs. Due to the subjectivity of LBP, six measures of LBP are captured: 1) any LBP, 2) LBP ≥ 5/10 pain rating, 3) LBP with medication use, 4) LBP with healthcare provider visits, 5) LBP necessitating modified work duties and 6) LBP with lost work time. Workers have thus far been enrolled from 30 different employment settings in 4 diverse US states and performed widely varying work. At baseline, workers undergo laptop-administered questionnaires, structured interviews, and two standardized physical examinations to ascertain demographics, medical history, psychosocial factors, hobbies and physical activities, and current musculoskeletal disorders. All workers’ jobs are individually measured for physical factors and are videotaped. Workers are followed monthly for the development of low back pain. Changes in jobs necessitate re-measure and re-videotaping of job physical factors. The lifetime cumulative incidence of low back pain will also include those with a past history of low back pain. Incident cases will exclude prevalent cases at baseline. Statistical methods planned include survival analyses and logistic regression.

**Discussion:**

Data analysis of a prospective cohort study of low back pain is underway and has successfully enrolled over 800 workers to date.

## Background

Estimates of the lifetime incidence of low back pain (LBP) in European and American countries vary from 49% to 70% [1-5]. Estimates of the point prevalence for LBP in western countries also vary somewhat from 12% to 30% [1,2,4,6]. When categorized by severity, Frymoyer et al. [3] found that 46.3% had moderate LBP and 23.5% had severe LBP. LBP is one of the most frequent and disabling conditions affecting workers in their productive years [7,8]. Recurrences are frequent [5,9-11]. LBP affects quality of life [12]; job selection [13]; and is a major reason for early retirement and disability pensions [14]. In the context of workers’ compensation, back claims represent 16% of total claims, but a disproportionate 33% of total claim costs [15,16].

Many investigations have reported higher incidence or severity of LBP among workers in heavy physical jobs than those with less strenuous jobs [17-23]. While multiple studies have identified individual job physical exposure variables (such as trunk flexion, twisting, weight of the object, etc.) as risk factors [24,25], it is believed that it is the combination of job physical exposure variables that subject a worker to high levels of biomechanical, physiological and psychophysical stresses [26-29].

Several ergonomic job evaluation methods have been developed to study biomechanical, physiological and psychophysical stresses from manual materials handling tasks. These methods include: i) the National Institute for Occupational Safety and Health (NIOSH) revised Lifting Equation [26,27], ii) maximum acceptable weights and forces [28], iii) static strength requirements of a job [30], iv) biomechanical models to estimate low back compressive and shear forces, [30-35], v) energy expenditure models to estimate whole body fatigue [29,36-38]; vi) statistically driven the Lumbar Motion Monitor model [39]; and vii) the State of Washington checklist. Several studies have shown that these models are associated with increased risk of LBP [19,38-46]. Yet, none of these methods have been fully validated with prospective cohort studies [47]. The vast majority of these studies have been (i) cross-sectional, (ii) have studied only job physical factors without controlling for potential individual and psychosocial confounders, and (iii) might have underestimated incidence of LBP by relying on relying on OSHA 300 logs for reported cases of LBP.

This prospective cohort study’s hypothesis is that there is relationship between quantified ergonomic factors and subsequent risk of low back pain after controlling for major risk factors and well-established confounders.

## Methods/design

This study is approved by the Institutional Review Boards of the University of Wisconsin – Milwaukee, the University of Utah and Texas A&M University (#03.02.059 and 11889 respectively).

**Figure 1:**
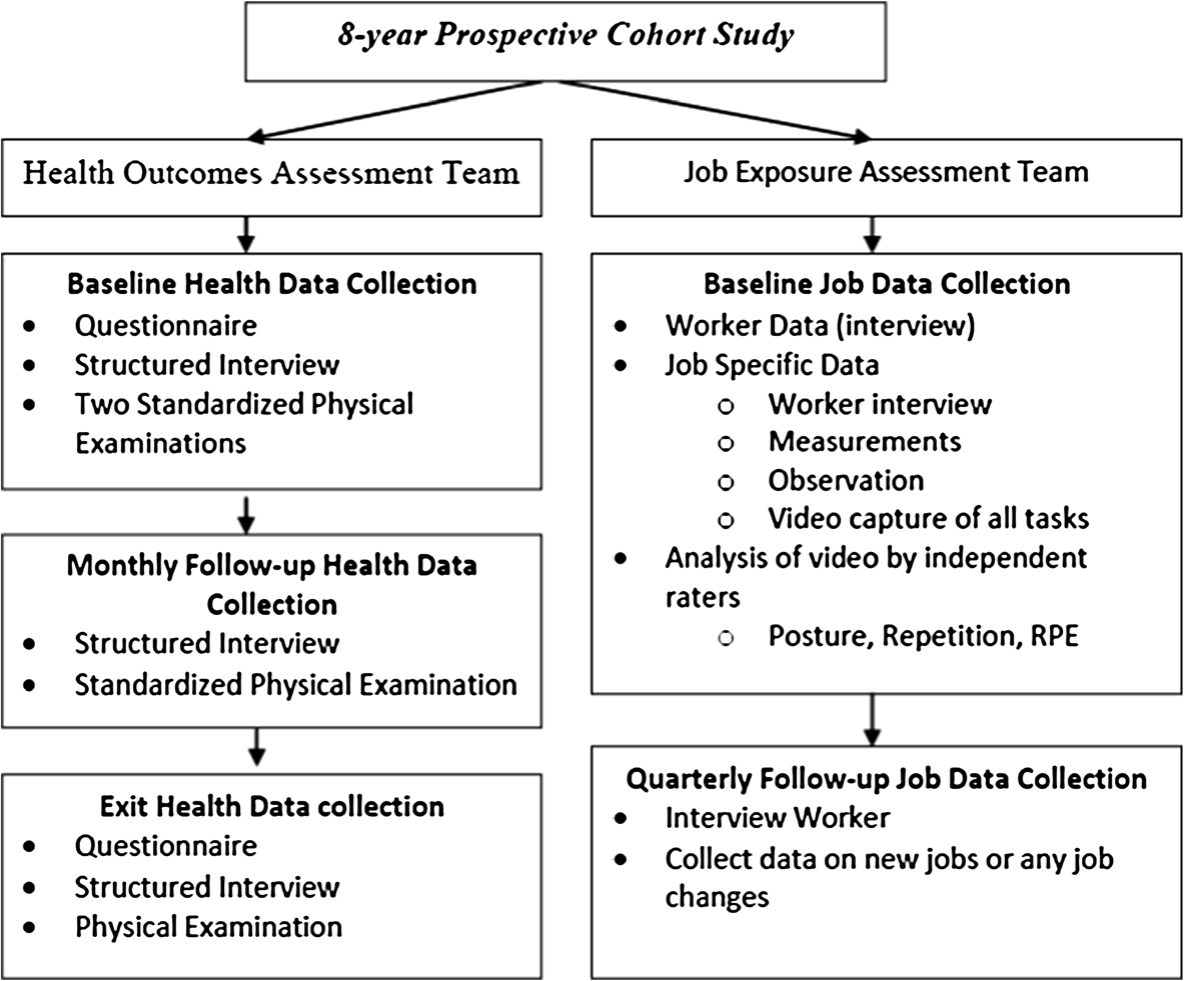
Data Collection Sequencing.

The design is a prospective cohort study. See Figure 1 for sequencing of data collection activities.

Workers for the study are recruited from 30 employers with 28 diverse production facility types located in Illinois, Texas, Utah and Wisconsin, USA. These employers include: (i) printing operations, (ii) toilet seat manufacturer, (iii) poultry processor, (iv) metal automotive parts manufacturer, (v) glass window & door manufacturer, (vi) repackaging operations, (vii) plastic parts manufacturer, (viii) metal parts manufacturer, (ix) brewery, (x) salt manufacturer and distributer, (xi) soft drink distributor, (xii) painting products, (xiii) electric lighting manufacturer, (xiv) lawnmower manufacturer, (xv) small engine manufacturer, (xvi) commercial printer, (xvii) warehouse/shipping/receiving, (xviii) airbag manufacturer, (xix) cabinetry manufacturer, (xx) alcohol distributor, (xxi) warehouses with grocery order selectors, (xxii) medical products manufacturer, (xxiii) apparel manufacturing, (xxiv) metal fabrication, (xxv) garage door manufacturer, (xxvi) office chair manufacturer, (xxvii) pharmaceutical, (xxviii) cosmetics manufacturer. Participating workers are paid regular wages by their employers. No incentives are paid for participation. Participants sign informed consent documents (Figure 1).

The goal is to enroll one-third of workers into each of three (low, medium and high) low back job physical demands groups. Eligible workers are required to be: (i) at least 18 years of age (due to job instability with youth), (ii) able to give informed consent, (iii) have no plans to retire or leave their employer within four years, (iv) able to speak either English or Spanish, and (v) free of major limb deformities and/or substantial amputations. Supervisors, maintenance/mechanics, and forklift truck drivers are not eligible due to probable frequent and unpredictable changes in job physical exposures as well as difficulty in efficiently videotaping these workers. However, workers transferring from eligible positions to office work or maintenance are subsequently followed for development of LBP.

The Health Outcomes Assessment Team is responsible for collecting LBP data, demographics, hobbies, physical activities outside of work, psychosocial and medical history data (see Additional file 1: Appendices A.1 – A.5). The Job Exposure Assessment Team is responsible for collecting job physical exposure data (Additional file 2: Appendices B.1 – B.10). These two different teams of researchers are blinded to each other.

### Baseline health outcomes data collection

After consent is obtained, the Health Outcomes Assessment Team administers questionnaires (see Additional file 1: Appendices A.1-A.5) and structured interviews (see Additional file 1: Appendix A.2) at baseline. The questionnaire and structured interview are computerized with skip sequences to speed administration and eliminate inappropriate questions (e.g., pregnancy for males or detailed questions on medical treatment when there had been no musculoskeletal disorder). It is believed that computerization would also improve data quality by ensuring standardized responses to questions and eliminating out-of-range responses. Both electronic and paper versions of the questionnaire are available in English and Spanish. Translators are used for questionnaires, structured interviews and examinations as required. Data were exported to a SAS database for management and analyses [48].

The questionnaire particularly includes medical health items and psychosocial factors (n = 30 items, see Additional file 1: Appendix A). Specific content areas include: (a) demographics (e.g., age, gender, and history of maximum body weight), (b) frequencies and durations of hobbies and outside of work activities, (c) medical history including diabetes mellitus, thyroid disorders, high blood pressure, high cholesterol, musculoskeletal disorders, inflammatory arthritis (including rheumatoid arthritis), and other relevant diseases, (d) psychosocial questions (e.g., depression, job satisfaction, family problems, supervisory and coworker support), and (e) other questions (e.g., sleeping patterns, smoking, alcohol consumption, family history of sciatica). The questionnaire also has a variety of psychosocial questions (44 items) including individual questions, Modified APGAR, and a subset of questions from Zung Depression scale are employed, as well as a composite tension-edge-nervous scale. There is a total of 44 items that are generally classified as psychosocial, ten of which are work organizational factors and some of these factors are assessed by the Job Exposure Assessment Team (see below). There are 7 items on job-related psychosocial factors (e.g., co-worker support) and 18 items on personal psychosocial factors (e.g., depression, anxiety). Additional psychosocial questions, pertaining to family problems, were added in 2009. Other factors could not be included due to practical requirements to balance inclusion of factors against the significant increased time commitments to complete such instruments. Excessive time for completion is a limiting factor for companies’ participation.

A structured interview is administered by either Physical Therapists or Occupational Medicine Residents and includes a survey of symptoms required for diagnostic purposes (n = 483 items) (see Additional file 1: Appendix A.2 for paper version). The structured interview also utilized a body diagram to help localize symptoms (see Additional file 1: Appendix A.3). Symptoms that are current as well as in the past month include (a) low back pain (b) thoracic spine pain, (c) neck pain, (d) lower extremity pain, (e) tingling and numbness in the lower extremity. Additional questions include a history of specific disorders such as sciatica.

A low back symptoms diagram is then completed by the worker for symptoms at the time of enrollment (see Additional file 1: Appendix A.2). To facilitate both of the two subsequent physical examinations and diagnostic impressions, the computer program displays a summary of the body parts that have been symptomatic in the prior month.

Workers then undergo the first of two standardized physical examinations by the same individual who administered the structured interview (see Additional file 1: Appendix A.2). Examinations include (a) observation, (b) inspection of the back, (c) palpation, (d) range of motion, and (e) specific examination maneuvers. Health team members are trained on the standard examinations through review of the videotape and practice in small group sessions until proficiency and consistency in accordance with the protocol are demonstrated. All maneuvers are performed in the first examination regardless of whether symptoms are present or not. These findings are recorded on paper to integrate information for the second physical examiner’s review (see Additional file 1: Appendix A.3).

The second physical examination is performed by board-certified Occupational Medicine physicians. There are two main information sources used to begin that examination: the symptoms summary page from the structured interview (above) and the results of the first examiner’s physical examination tests. The purpose of this examination is to confirm positive findings and to evaluate pertinent negatives. Additionally, this examination results in a diagnostic impression irrespective of the specific physical examination results and case definitions (see below).

Height and weight are measured in stocking feet in metric units (see Physical Examination form in Appendix A). These are used to calculate body mass indices (BMI). If the weight exceeds 200kg, two scales are used simultaneously with the sum of the two scales recorded. Blood pressure and heart rate are obtained in a seated position after a minimum of 5 minutes of rest, and most often after 20 minutes of rest (after completing the questionnaire). Automated cuff measures are utilized (Omron HEM-780).

### Follow-up health outcomes

After completing the baseline health outcomes evaluations, workers are placed into a monthly follow-up system. Each month, a member of the health outcomes assessment team assigned to that plant visits that facility. That team member conducts a brief interview with each of the participating workers. These interviews typically last for less than five minutes and are conducted at the worker’s workstation. All interviews are conducted using laptop computers to enable referencing of the health status from previous month except at one facility that does not allow electronic devices on the production floor. In that case paper copies of the interview are used, with the previous month’s data printed on the data collection forms.

The purpose of monthly interview is to (i) determine current health (LBP) status and (ii) maintain worker enthusiasm in the study. By referencing the worker’s health data from the last follow-up, workers are asked if their pain and/or numbness and tingling have changed or resolved and how many days ago. If the pain did not go away, the workers are asked to provide a pain rating and percent of days they had pain since last follow-up. The workers are next asked if they developed any new pain in either the low back and/or legs since the last follow-up. If new pain (or numbness and tingling) is identified, the workers fill out the low back pain diagram (Additional file 1: Appendix A) to document the location(s) of pain, rate the intensity of pain on the pain scale, are asked when did the pain start (date), how many days they had pain, what they believe was the cause of the pain (unsure, accident outside of work (slip/trip/fall, motor vehicle accident, etc.…), other cause outside of work (poor sleep/mattress, single lift, multiple lifts, single push, multiple pushes, single pull, multiple pulls, other), accident at work (slip/trip/fall, motor vehicle accident, etc.…), work (anything job related, but *not* previously classified as a work accident), and relapse or aggravation of previously reported pain. The workers are asked if they had seen a (i) chiropractor, (ii) physician, and/or (iii) a physical or occupational therapist. They are also asked if they have (i) undergone a spine surgery, (ii) given an injection (such as steroid injections, etc.…), and/or (iii) used a back brace. In addition, a variety of other treatments are captured as they relate to low back pain treatment history. These include: (i) non-steroid anti-inflammatory drug (NSAID), (ii) over the counter medications, (iii) prescription medications (not NSAID or narcotic), (iv) narcotic killer prescription medication, (v) aerobic exercises, (vi) strengthening exercises, and (vii) stretching exercises.

Lastly, workers are asked to describe any job changes they had since the previous monthly interview. The objective is to assist the Job Physical Exposure teams to determine if additional follow-up of job is required. Job changes questions include (i) moving to a new job/line, (ii) using new equipment/tools, (iii) an increase or decrease in production rate, (iv) a change in work hours (≥ 5 hours per week), and (v) any “other” changes. Workers are also asked when these changes occurred.

### Baseline job physical exposure data

Job physical exposure data, as well as some work organizational factors, are collected at the facilities of the participating companies by Job Exposure Assessment Team members (hereafter, analysts). Analysts contact the worker and interview them to determine the worker’s job(s) prior to measuring and videotaping exposure. Field data collection is performed often using teams of three analysts. Teams visit one facility per day, and collect data for 8–10 hours per day. One member video records the job, a second member takes physical measurements, and the third member records data. All jobs performed by participating workers are recorded on digital videotape using hand-held video cameras. Jobs with cycle time ≤ 2m are recorded for at least ten cycles and jobs with cycle time > 2m are recorded for 15 to 45m, often ensuring at least one complete cycle recorded. Video is ideally taken perpendicular to the sagittal plane to facilitate biomechanical analyses performed in laboratories. In those cases where there is trunk lateral bending or twisting is involved videos are taken from different angles. Tasks with cycle time less than or equal to 2 min were videotaped for at least three cycles from each of three angles, and tasks with cycle time > 2 min were videotaped for at least 5 min from each of the three angles (Table 1).

**Table 1:** Physical exposure at the worker level (measurements/observations in the field)

**Exposure Type**	**Measurements**
General	Job title, department, shift length
Pace	Self-paced, line-paced, or piece work
Job rotation	No. of jobs, duration of each job, title of each job
Prior work experience	Title, years on each job, and worker’s Rating of Perceived Exertion (RPE, [49]) for low back and each job
Second job outside facility	Title, years on second job, and worker RPE for low back.
Strength	Grip, lateral pinch and 3-point pinch for dominant hand
Fatigue	Overall worker low back RPE 30minutes after the beginning and 30min before the end of the shift.

Quantitative measurements included: (i) object weights (using digital platform scale), (ii) pushing and pulling forces (force gauge, model # CSD250, manufactured by Chattilon), (iii) horizontal and vertical hand locations for each hand (tape measures) (iv) push/pull and walk/carry distances (rolling tape measuring device), and (v) duration of task activities (digital stopwatches). Forces and exertions are also rated subjectively using the Borg CR-10 Scale [49]. Using the Borg CR-10 scale, workers are asked to rate how hard the task is on their low back. At most sites analysts also provide their Borg CR-10 rating for the same tasks.

Baseline data collection is broken into two major components: (i) position specific data collection (Additional file 2: Appendix B.1) and (ii) job specific data collection (see Additional file 2: Appendix B.3) In this study, *position* refers to the worker’s overall activities in a day (workshift). *Job* refers to specific, but unique, activities performed by the worker for a certain number of hours in a given day (see Figure 1). A position can be comprised of a single job or multiple jobs (e.g. job rotation). For example, a worker may perform three different jobs in a given day: packaging of light fixtures and components into a box for 5 hours (Job 1), palletizing finished boxes of light fixtures for 2 hours (Job 2) and moving pallets full of boxes to the staging area for 1 hour (Job 3). Each of the three jobs exposes this worker to different levels of biomechanical stresses. Further, each of the three jobs requires lifting of different weights (light fixture components weigh from 1 kg to 22 kg). Each time a lift is performed the horizontal location of hands, vertical location of hands, body posture, etc. may vary. In addition certain jobs may require combinations of lifting, lowering, holding, pushing, pulling, carrying, and walking. In this study, each physical exertion is characterized by nature of the exertion (lifting, lowering, pushing, pulling, etc.).

#### Position specific job physical exposure data

Position specific data are collected to determine all different jobs performed by the worker and related information (see Additional file 2: Appendix B.1). Position data include: (i) department and worker title, (ii) shift starting and ending time, (iii) different jobs performed, (iv) hours worked on each job, (v) job pace (self-paced, line-paced, or piece rate), (iv) days worked per week, (v) overall rating of perceived exertion (RPE) for the lower back (Borg CR-10 scale) [49] at the beginning and end of the shift, vi) prior work experience (job title, duration in years and recalled RPE for the lower back [49] and (vii) having a second job (brief description, duration in years, hours/week, and low back RPE [49].

Regarding prior work experience, the first position listed is the “Current” position the employee holds. Previous positions are listed until the total previous employment duration sums to 10 years, or 5 previous (6 total, including the current) positions are recorded, whichever occurs first. Workers are asked to provide a corresponding Borg CR-10 rating for low back for each of the positions listed.

With regard to their primary position, the worker is asked to provide RPEs for the level of physical stresses they feel in their lower back at the beginning of their work shift (about 30 minutes after they started their typical work day) and at the end of their work shift (about 30 minutes before the end of their typical work day). This information is gathered to attempt to estimate the accumulation of fatigue as a result of performing the position activities.

Secondary employment information includes type of work, number of years worked on second job, hours/week worked on second job and Borg CR-10 rating for low back from second job.

#### Job specific job physical exposure data

Data are collected for each job performed by a worker using Job Specific Data forms (Additional file 2: Appendix B.3). Analysts’ general observations include: (i) use of back belt, (ii) type of floor surface (normal, uneven, slippery), (iii) use of anti-fatigue mat, (iv) use of “insoles” in shoes, (v) exposure to whole body vibration, (vi) workspace (open, obstructed), (vii) plant temperature (measured), and (viii) plant humidity (measured). Workers are asked to estimate what percentages of their time they spend on (i) manual material handling, (ii) assembly operations, (iii) paper work, (iv) fork-lift truck riding, and (v) resting/waiting. For each of these activities they are asked whether they perform these activities in seated posture or standing. For assembly tasks, they are asked whether it is light or heavy assembly. Workers are asked to provide a RPE rating for low back for each of these tasks. Analysts provide their own RPE ratings for the low back (Borg CR-10 scale) after observing each of the above activities as applicable. Workers are asked to provide percentages of work shift spent with back bent > 20° and squatting. Workers are also asked to identify the most stressful task they perform during a work shift and provide a RPE rating for low back for that task. Analysts provide their own RPE rating after observing that task.

Job Specific data are collected for each job performed by a worker during a work shift. A job is further broken down into (i) lifting/lowering, (ii) pushing/pulling and (iii) walking/carrying tasks. For each lifting and lowering task measured data in the field include: nature of the task (lift or lower), weight of the object, horizontal locations of hands from their respective ankles [27] at the origin and the destination of lift/lower, vertical locations of each hand at the origin and destination of lift/lower and box width. For pushing/pulling tasks measured data in the field include nature of task (pushing or pulling), initial force, sustained force, hand height, distance pushed/pulled and duration of push/pull in seconds. Field measurements for walking and carrying tasks include nature of task (walk or carry), weight carried, distance of carry/walk and duration of carry/walk in seconds. These data are collected for each task (i.e., each physical exertion performed by a worker).

#### Data extraction from videos

Other job data are obtained by reviewing videotapes including observation time, cycle time and frequencies of different lifts, lowers, pushes, pulls, walks and carries. Videotapes are analyzed frame by frame. Each lift, lower, push and pull task is analyzed to determine body joint angles including trunk flexion, axial rotation and lateral bending angles for biomechanical analyses (University of Michigan 3D Static Strength Prediction Model (3D SSPP) ver. 5.0.8). For lifting and lowering tasks these angles are determined at the beginning and end of lift/lower. In addition, lifting and lowering tasks are analyzed to determine: (i) nature of task (lift or lower), (ii) number of lifts/lowers for each lift/lower characterized by its weight, horizontal and vertical locations [27], (iii) asymmetric angle [27], (iv) type of grasp (good, fair, poor; [27]), (v) number of hands used (right, left or both), (vi) lifting technique (stoop, semi-stoop, squat, side, stand), (vii) nature of object (box, bag, etc.), and (viii) object length and height. Similarly, pushing and pulling activities are analyzed to determine (i) nature of activity (pushing or pulling), (ii) type of object pushed/pulled (four-wheel cart, two-wheel hand truck, pallet jack, box, other), (iii) number of hands used to push/pull (right, left or both), body posture (lean forward, lean back, lean to a side, standing), and (iv) number of pushes/pulls characterized by initial force, sustained force, hand height, distance and pushing/pulling time. Video analysis for walking/carrying included (i) type of activity (walking or carrying), (ii) number of hand used (left, right or both), (iii) technique for carrying (against waist, thighs, side), (iv) type of object carried (box, bag, other), and (v) number of walks/carries.

### Follow-up job physical exposure data collection

Trained ergonomics analysts visit each worker every three months to assess job physical exposure changes. The analysts also carry a computerized position form with them showing jobs the worker was performing as of the last visit (3 months prior). The analyst inspects all the jobs listed and determines if there are any changes to the jobs. If either a worker or a supervisor reports a change in the job or the analyst believes that the job has changed, physical exposures are reassessed using the same protocol utilized at baseline.

### Job rotation and jobs with multiple tasks

Many workers in this study have job rotations, i.e., they perform two or more jobs during a workshift. Practically all jobs consist of multiple tasks where either nature of the exertion (lifting, lowering, holding, pushing, pulling, etc.) changes or one or more task parameters such as force, weight, horizontal location of hands, vertical location of hands, body posture, etc. change with each exertion (multiple task jobs). Traditionally, (i) peak/maximum exposure [19,39], (ii) simple average [39], (iii) time-weighted average (TWA) [50,51], (iv) frequency-weighted average [52,53] and (v) cumulative exposure [54,55] have been used to quantify and assign exposure to worker. These approaches, are believed to either underestimate or overestimate job physical exposure [56-58], and may erroneously classify unsafe jobs as safe jobs and vice-versa [58]. Using average and TWAs to represent force or weight dilutes the effect of intermittent, hazardous exertions. Conversely, peak force over-estimates the true hazard as often it assumes all exertions are at that level. An appropriate method for assigning job physical exposures at the worker level has not been clearly identified in literature. A few studies suggest that physical exposure when quantified as peak exposure at the task level may best discriminator LBP risk in the workplace [19,43]. Another approach is to use the Composite Lifting Index (CLI) to quantify biomechanical exposure from lifting/lowering tasks at the job level. However, CLI is calculated at the job level and does not take into account job rotation.

### Index based methods for determining physical stresses from a job

For each physical exertion data from field measurements and videotapes are combined together. These data are used to calculate the following job physical exposure indexes:

•Lifting Index (STLI) for each task (see Figure 1) using the Revised NIOSH Lifting Equation (RNLE) [27];

•Composite Lifting Index (CLI) for each job using the Revised NIOSH Lifting Equation (RNLE) [27];

•Compressive and shear forces for each task using the 3-D Static Strength Prediction Program (3D SSPP, Center for Ergonomics, University of Michigan);

•Strength requirements of each task (% capable males and females) using the 3-D Static Strength Prediction Program (3D SSPP, Center for Ergonomics, University of Michigan);

•Load moment on low back (Weight of the object × load moment arm).

•Fatigue (RPE at the end of shift – RPE at the beginning of shift).

### Assigning exposure at the worker level

**Figure 2:**
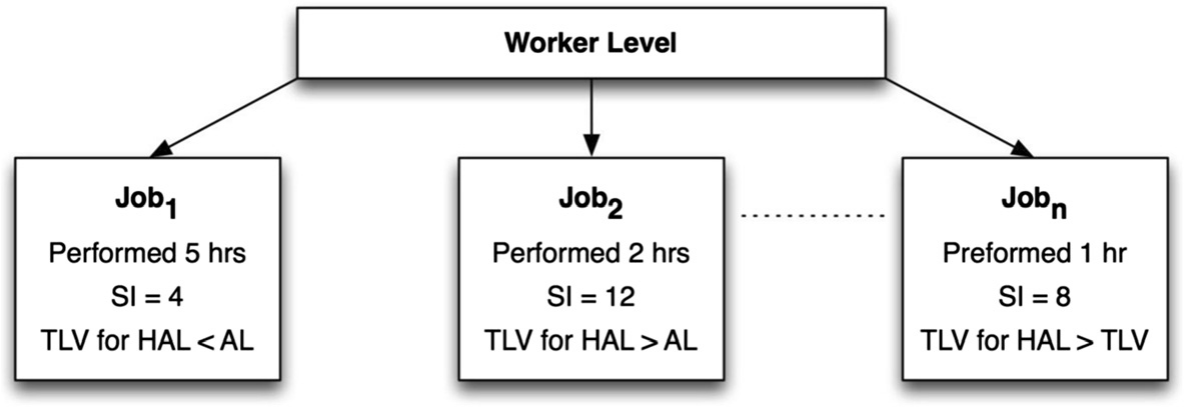
**Example of a worker’s job for illustrating exposure classification.** Job1 represents the longest job performed in the day and thus it is the typical exposure for the Strain Index (SI) and Threshold Limit Value for Hand Activity Level (TLV for HAL). Job2 represents the peak exposure for the SI and Jobn represents the peak exposure for the TLV for HAL as it has the highest threshold limit value, which exceeds the TLV.

Due to lack of guidance in the literature for assigning physical exposure at the worker level, we plan to use several different indexes of physical exposure. These include:

1. Peak Single Task Lifting Index (peak STLI), i.e., Lifting Index (LI) from a task that produces the highest (LI).

2. Peak Composite Lifting Index (Peak CLI), i.e., Composite Lifting Index from a job that produces the highest CLI (see Figure 2).

3. Typical Composite Lifting Index (Typical CLI), i.e., CLI from a job that is performed for the largest percentage of a workshift (see Figure 2).

4. Cumulative Lifting Index (CULI), i.e., combining CLIs from all jobs performed by a worker using an incremental stress approach similar to that used for computing CLI from STLIs.

5. Peak compressive force, i.e., compressive force from a task that produces the highest compressive force on low back.

6. Peak load moment, i.e., load moment from a task that produces the highest load moment.

7. Minimum percent capable population (from 3D SSPP), i.e., worker gender specific percent capable population from a task that produces the minimum percent capable population (Figure 2 and Table 2).

**Table 2:** Physical exposure at the job level (measurements/observations in the field (m) and from videotape analysis (v))

**Variable**	**Analyst**	**Worker**
Cycle Time (min)	• SI definition (v)	
Force	• DUE force rating each job	• DUE force rating each job (Borg CR-10)
	o Peak force (m)	o Peak force (m)
	o Typical force (m)	o Typical force (m)
	• Overall DUE expert force rating1 (v)	• Matching force
	• Object/tool weight and Center mass offset (m)	o Grip force (m)
	• Pushing/pulling force (m)	o Pinch force (m)
		o Thrust force (m)
Repetition	• HAL Rating (v)	
	• No. of exertions/min (SI) (v)	
	• No. exertions/minute at each force level2 (v)	
	• Speed of work (SI) (v)	
%Duration of Exertion	• % Duration of exertion (SI) (v)	
	• Duration per exertion for each force level (s/exertion) (v)	
Exposure/day (hours)	• Worker interview(m)	
Hand/wrist Posture	• Posture categories3	
	o Wrist flexion: <30°, 30°-50°, >50° (v)	
	o Wrist extension: <30°, 30°-50°, >50° (v)	
	o Ulnar deviation: <10°, 10°-25°, >25° (v)	
	o Radial deviation: <5°, 5°-25° (v)	
	• No. of exertions in each category (v)	
	• % of cycle time in each category (v)	
	• Peak force posture categories (v)	
	• Hand/wrist posture each task (SI) (v)	
	• Overall SI posture (v)	
Elbow Posture/Forearm Rotation	• Extension < 70° and > 135°	
	o No. of exertions (v)	
	o % cycle time (v)	
	• Forearm rotation	
	o (Neutral, prone, supine) (v)	
	o % of cycle time with forearm rotation > 45° from neutral (v)	
Grip/pinch	• Type of grasp/pinch4 (v)	
	• Grip/pinch span (v)	
	• % cycle time in each type of grasp/pinch (v)	
Localized Mechanical Compression	• Body part	
	• Category (Negligible, moderate, severe) (m)	
	• No. of exertions (v)	
	• % of cycle time (v)	
Hand as hammer	• Category (Negligible, moderate, severe) (m)	
	• No. of exertions (v)	

### Case definitions

Case definitions utilizing the history and structured interview results were defined. No acute injury events whether at home or work (e.g., slip, trip, fall) will be included in the analyses for risks of diseases although they will be summarized. Primary analysis will be for first time LBP lasting more than 24 hours. Additional analyses including recurrence will also be performed. A case-free interval of at least 3 months will be required prior to eligibility to develop a recurrent case.

The primary case definitions for low back pain are: 1) any degree of LBP, 2) LBP with pain greater than 5/10, 3) LBP with medication use, 4) LBP with healthcare provider visits, 5) LBP necessitating modified duty and 6) LBP with lost work time.

### Statistical analyses

Key analytic objectives in this study include: 1) Determination of prevalence for low back pain, 2) Calculation of incidence rates, 3) Evaluation of risk factors for LBP, 4) Evaluation of interactions between various risk factors, 5) Assessment of the performance of existing ergonomic models, and 6) Building models for predicting risk(s) of LBP. We outline below the general strategies that we will employ in the statistical analyses. Additional details will be provided in the manuscripts addressing each objective.

#### Preliminary analyses and transformations of variables

Univariate summaries of exposure variables, outcomes, and covariates will be provided and quantitative variables will be depicted graphically using histograms, kernel density curves, and normal probability plots. These summaries will be reviewed by the study team members who will apply statistical (e.g., roughly equal numbers of patients in each category) and clinical criteria to define cut points for subsequent categorical analyses and to determine appropriate transformations of continuous exhibiting heavy deviations from normality.

We will also summarize pairwise relationships among blocks of exposure variables and covariates representing the common domains using matrices of appropriate association measures (e.g., Pearson correlations for continuous variables, odds ratios for binary variables), along with variable clustering methods [48]. The patterns of association will be reviewed by the study team to remove highly correlated redundant variables, or replace highly correlated factors with averages or first principal component scores. These steps will reduce multicollinearity and the complexity of subsequent statistical models, and will be completed prior to the conduct of analyses relating health outcomes to exposure variables.

#### Assessments of prevalence and incidence in the study population

To characterize the cohort, point prevalence in the study population will be estimated for three measures of LBP (e.g., any LBP, LBP > 5/10, LBP with medication use) at baseline and exact 95% binomial confidence intervals computed. Lifetime prevalence estimates will also be estimated at baseline for: 1) all LBP, 2) LBP greater than 5/10, 3) LBP with medication use, 4) LBP with healthcare provider visits, 5) LBP necessitating modified duty and 6) LBP with lost work time.

Incidence rates for new occurrences of the measures of LBP will be estimated as the ratios of the numbers of occurrences of each LBP type to patient-years of LBP follow-up. As such, repeat occurrences of LBP in the same workers will be counted when computing incidence rates. Subjects will be right censored at loss-to-follow-up and interval censored during periods at which new occurrences of LBP are not ascertained. Nonparametric estimates of cumulative incidence curves for interval censored data [59] will be used to display cumulative incidence of first new occurrences of each of the indicated measures of LBP since baseline.

#### Regression and covariate adjustment

Regression models will be used to relate indicators of LBP and other health outcomes to potential exposure variables and covariates. Logistic regression will be employed for analysis of prevalence of LBP expressed as presence of particular LBP measures, ordinal logistic regression for ordered categorical outcomes, negative binomial regression with robust covariance matrix estimates for count data (such as number of lost or restricted work days in a designated time interval), and survival analysis methods for incidence of new occurrences of LBP.

Associations between risk factors and health outcomes will initially be evaluated using the univariate regression methods appropriate to each outcome. Key risk factors include job physical factors (e.g., object weight, pushing force, pulling force, lifting frequency, posture), as well as the revised NIOSH Lifting Equation and back compressive forces. We will also consider models relating health outcomes jointly to two more risk factors with interaction terms to evaluate if the association of a health outcome with one risk factor depends on the level of one or more other risk factors. In particular, combinations of job physical factors, object weight, lifting frequency, and posture will be evaluated. We will also assess interactions between individual risk factors (particularly psychosocial factors, smoking and obesity) and combinations of job physical demands.

In subsequent analyses, associations between health outcomes and risk factors will be evaluated after adjustment for covariates selected to control for known sources of confounding. For this purpose, potential covariates (e.g., worker demographics, hobbies and physical activities outside of work, psychosocial factors, baseline prevalence of LBP other than the disorder being analyzed and medical history will be grouped and evaluated for association with the health outcome variable prior to their addition to models with the target risk factor(s). Covariates will be selected using guided stepwise procedures in which essential covariates are designated for inclusion based on subject matter considerations and others are added if found to be independently predictive of the outcome.

To the extent possible, we will retain the variable transformations and categorizations defined in the preliminary analyses prior to regression analysis (see above). However, regression diagnostics appropriate to each regression methodology [60,61] will be used to screen for violations of modeling assumptions, including deviations from linear relationships, interactions not specified in the base statistical models, and non-proportional hazards for event outcomes. Substantial deviations from modeling assumptions will be documented, and models augmented as necessary to account for model deviations. In particular, we will employ cubic smoothing spline methods to account for clear nonlinear relationships.

We note that while this cohort study incorporated detailed measures of job stress as well as substantial covariate information, we do not view the collection of covariate information as sufficient to control for all confounding, particularly time dependent confounding. Accordingly, results of regression analyses will be interpreted as characterizing association which may be informative for underlying causal effects, but not as direct estimates of causal effects. In part for this reason, we will emphasize regression methods rather than formal causal effect estimation methodologies such as propensity matching.

#### Predictive models

For assessment of the predictive performance of existing ergonomic models (e.g., revised NIOSH Lifting Equation, back compressive forces), the incidence data will serve as the reference against which the operant characteristics such as sensitivity and specificity will be analyzed [62,63]. We will also employ time-dependent ROC methods to characterize relationships between incidence over time with prior ergonomic assessments. We further evaluate existing ergonomic models by refitting coefficients of their component terms and addition of other terms in new predictive models derived from the present cohort, with subsequent comparisons of prediction accuracy between existing models and new models evaluated using 10-fold cross-validation [64].

A statistically driven model accounting for all weights, forces, horizontal locations, vertical locations, frequencies of exertion, trunk flexion angles, will be developed through this work. As this is still to be developed, specific details on decision logic and model building will be published subsequently, with full transparency of analyses used to develop the model disclosed at that time.

#### Survival analysis

As described above, univariate and multivariable Cox regression analyses will be used to relate newly incident cases of LBP to ergonomic factors [e.g., revised NIOSH Lifting Equation, back compressive forces (see above)] as well as individual ergonomic variables (e.g., object weight, pushing force, pulling force, lifting frequency, posture) [65]. In general, will be consider both Cox models relating incidence of LBP over follow-up to baseline risk factors with interactions between baseline risk factors and follow-up time to assess longer-term implications of exposures at a given point in time, and time-dependent Cox models relating incidence of LBP to the most recent time-dependent measurements of exposure variables to assess short-term association of outcomes with risk factors. Separate proportional hazard regression models will be fit for each of the job physical exposure tools (revised NIOSH Lifting Equation, back compressive forces) as well as individual job physical exposure variables such as object weight and lifting frequency.

Additional analyses to model associations with events occurring more than once in the same individual (e.g., LBP that recurs 6 months later) are planned using the Andersen-Gill independent increment method [66] and other approaches. These other approaches involve fitting a basic proportional hazard model that ignores potential correlations to an appropriately define risk set, and then implementing a robust covariance estimate to adjust for correlation between events occurring in the same subject [67]. Random frailty models will also be considered to evaluate the extent of variation in hazards between workers after accounting for measured covariates [68].

#### Multiple comparisons

The issue of multiple comparisons looms large for cohort studies such as the present one in which multiple research questions are addressed, and multiple exposure and outcome definitions will often be considered when investigating specific questions. We plan to address distinct research questions on a comparison-wise basis, without adjustment for multiple comparisons [69]. The following strategies will be used to minimize risk of inflated Type 1 error when addressing specific research questions: 1) Adherence to documented decisions regarding category definitions and transformations of variables, 2) Development of analysis plans designating outcome variables, risk factors, covariate adjustment strategies and other analytic decisions prior to analyses for each research question, 3) Designation in analysis plans of a limited number of “primary analyses” that use uncorrected significance levels, 4). Differentiating exploratory analyses, sensitivity analyses, and other post-hoc analyses from pre-specified analyses and reporting them as such, 5) properly accounting for each term’s total degrees of freedom when applying flexible semi-parametric methods such as cubic splines, and 6) utilization of cross-validation to assess accuracy of predictive models. In select cases, formal multiple comparisons procedures will be designated in analysis plans when statistical power is sufficient and it is not plausible to define a single primary analysis for a particular research question.

#### Missing data

The results of regression models will generally remain valid so long as the missing data pattern satisfied the missing at random condition (MAR), which stipulates that the probability of missingness does not dependent on unobserved values of missing responses after taking into account the nonmissing data. Survival analysis methods will facilitate the use of available follow-up data in subjects who drop out of the study, under the assumption of noninformative censoring. We will employ multiple imputation in analyses in which substantial portions of data are missing and the MAR assumption is suspect. Multiple imputation will be carried out using full likelihood Markov chain Monte Carlo (MCMC) sampling [70] assuming multivariate normality when outcomes are approximately normally distributed, and using fully conditional imputation otherwise [71].

## Discussion

A large, multi-center prospective cohort study is underway to quantify risks of low back pain and to assess the performance of existing ergonomic job evaluation methods. This cohort study is addressing numerous weaknesses of prior research studies including use of: 1) prospective methods, 2) multi-center with four diverse states, 3) computerized data collection methods of questionnaires and structured interviews to assure data collection, 4) standardized physical examinations that include one comprehensive examination and one symptom focused examination, 5) ability to exclude pre-existing or prevalent cases at baseline, 6) blinding of Health Outcomes Assessment and Job Exposure Assessment Teams, 7) monthly follow-ups of the cohort individualized quantification of job physical exposures, 8) heavy reliance on objective measures of exposure, 9) methods to account for job rotation and multiple task analyses, 10) careful case definitions, and 11) plans to evaluate interactions between and among job, individual and psychosocial factors.

Subjects are being enrolled from 30 employers with over 800 subjects having been enrolled to date. The overall participation rate is not known as several plants invite workers to participate in a meeting from which enrollments ensue, thus the total target population in those plants is unknown. The highest participation rate in one plant with individualized enrollment processes is 96.0%. In plants enrolling in group meetings, approximately 75% of subjects attending those meetings enroll.

The cohort has been followed for several years. The success rate in contacting the cohort on a monthly basis has been calculated at 83.5%. The success rate of identifying reasons for absences is nearly 100%. Reasons for worker absences are tracked, and include: (i) vacation (most common), (ii) illness, (iii) leave of absence (e.g., funeral), and in a few cases (iv) absence due to surgery or treatment for a musculoskeletal disorder at any given observation period.

Study limitations include that workers are primarily from manufacturing environments, thus the results might not be applicable to other environments. Some of the commonly reported non-occupational risk factors are likely to be underpowered due to limited sample sizes for those conditions as this is a convenience sample that targeted one-third low, medium and high job physical demands. Also, the numbers of psychosocial questions was somewhat limited by the practical limits of time allowed by participating companies for enrollment of subjects and may be insufficient for some psychosocial variable domains.

## Competing interests

The authors have no competing interests.

## Authors’ contributions

AG serves as study Principal Investigator (PI), designed the study, is responsible for all phases of the project, serves as the lead for the Job Exposure Assessment Teams (JEATs) and helped draft the manuscript. KH helped design the study, serves as the PI for the University of Utah, leads the Health Outcomes Assessment Teams and drafted the manuscript. JSM serves as the PI for Texas A&M University. AG, DB, JK, AM, RS coordinate the ergonomic measurements and the Job Exposure Assessment Team’s activities. KH, JSM, GD, JF, EW coordinate the health outcomes measurements and the Health Outcomes Assessment Team’s activities. MT, GS, TG, XS, RH coordinate the data and statistical management team. All authors read and approved the final manuscript.

## Pre-publication history

The pre-publication history for this paper can be accessed here:

http://www.biomedcentral.com/1471-2474/14/84/prepub

## Supplementary Material

Additional file 1: Appendix A Health Assessments. Click here for file

Additional file 2: Appendix B Job Specific Data. Click here for file
