# Supplementary material for: Study protocol title: a prospective cohort study of low back pain
Source: BMC Musculoskelet Disord. 2013 Mar 7;14:84. doi: 10.1186/1471-2474-14-84 (PMC3599364; doi:10.1186/1471-2474-14-84)
Supplement: Additional file 1: Appendix A — Health Assessments. [file 1471-2474-14-84-S1.pdf]

APPENDIX A

A.1. Baseline Questionnaire ....
A.2. Structured Interview ....
A.3. Physical Examination ....
A.4. Monthly Follow-up ....
A.5. Exit Interview ....

A.1. Baseline Questionnaire

An Ergonomic-Epidemiologic Study of the Low Back: Baseline Questionnaire

Directions:

Please answer each question by pointing the arrow with the mouse and clicking with you index finger to either mark “yes” or “no” or to fill in a blank. If you need help or have any questions please ask one of our research assistants. We’re happy to help!

Code # ________________________
Date: _________________________

An Ergonomic-Epidemiologic Study of the Low Back: Baseline Questionnaire

1. Your Company’s name: ____

2. Department: ______________________________

3. Job Title: ___________________________________

4. Age (in tenths) _____.__ years (calculated on the computer)

5. Gender: ____Male ____Female

6. How long have you worked for <<company name>>? _____ years ______ months

7. How long have you worked at your current job? _____ years ______ months

8. Altogether, approximately how many miles do you travel in a car or bus on an average day? _____ miles

9. Altogether, approximately how many minutes do you spend traveling one way to your job on an average day? _____ Hours _____ Minutes

10. Do you walk on a regular basis **at least twice a month** outside of work? ____Yes ____No

    a. If yes,

Type of Exercise	Number of months per year	Average number of times per week	Average number of minutes each time
Walking	Months	Per week	Minutes

b. Do you do any other exercise outside of work (for example, bicycling, basketball, skiing, or swimming) at least twice a month? ____Yes ____No

If no, skip to question 11.

a. Which of the following types of exercise (outside of work) do you do?

Type of Exercise	Yes	No	Number of months per year	Average number of times per week	Average number of minutes each time
Aerobics, Jazzercise			Months	Per week	Minutes
Running, Jogging			Months	Per week	Minutes
Bowling			Months	Per week	Minutes
Bicycling			Months	Per week	Minutes
Swimming			Months	Per week	Minutes
Weight Lifting			Months	Per week	Minutes
Baseball			Months	Per week	Minutes
Basketball			Months	Per week	Minutes
Football			Months	Per week	Minutes
Soccer			Months	Per week	Minutes
Racquetball			Months	Per week	Minutes
Handball			Months	Per week	Minutes
Tennis			Months	Per week	Minutes
Snow Skiing or Snowboarding			Months	Per week	Minutes
Water Skiing or Wave Runner			Months	Per week	Minutes
Hunting			Months	Per week	Minutes
Fishing			Months	Per week	Minutes
Martial Arts (Karate, Judo, etc.)			Months	Per week	Minutes
Other (please list)			Months	Per week	Minutes
Other (please list)			Months	Per week	Minutes

11. Altogether, outside of work, approximately how much time in a usual day do you spend sitting down (e.g. watching TV, reading, using a computer, and/or going to the movies, etc…)? ______ hours_____ min. per day

12. Outside or work do you participate in any activities or hobbies (for example, gardening, snow shoveling, car maintenance or doing housework, etc.) on a regular basis, at least twice a month?

If no, skip to question 13.

Type of Hobby	Yes	No	Number of months per year	Average number of times per week	Average number of minutes each time
Yoga / martial arts / relaxation therapy			Months	Per week	Minutes
Gardening, Landscaping			Months	Per week	Minutes
Snow Shoveling			Months	Per week	Minutes
Maintenance (e.g. car or engine repair), Mechanical Work			Months	Per week	Minutes
Driving a motorcycle or ATV			Months	Per week	Minutes
Snowmobiling			Months	Per week	Minutes
Building and/or repairing furniture or woodworking			Months	Per week	Minutes
Remodeling or building a home			Months	Per week	Minutes
Housework (mopping, cleaning, dusting, etc)			Months	Per week	Minutes
Knit, sewing, needlepoint, crocheting. Or arts and crafts			Months	Per week	Minutes
Other (please specify)			Months	Per week	Minutes
Other (please specify)			Months	Per week	Minutes

13. Have you ever been told by a physician that you have any of the following:

a. Diabetes: ___ Yes ___ No
Approximately how many years ago was this diagnosed? _________Years

With which of the following are you treating the Diabetes?
____ Insulin
____ Pills / Oral Agents
____ Both Insulin and Pills
____ Diet only (no insulin or pills)

b. Chronic Fatigue Syndrome? ___ Yes ___ No

c. Irritable Bowel Syndrome? ___ Yes ___ No

d. Thyroid problem: ___ Yes ___ No

e. Gout: ___ Yes ___ No

f. Kidney Failure: ___ Yes ___ No

g. High Blood Pressure: ___ Yes ___ No

h. High cholesterol (Laboratory test result over 200 mg/dL) ___ Yes ___ No

i. Hernia (Abdominal or Inguinal hernia, **does not** include herniation of disk in neck or back):
_______Abdominal (Belly Button)
_______Inguinal (Groin)
_______Both

14. Have you ever been diagnosed with any other problems?
a. Other:__________________________(please specify) ___ Yes ___ No

b. Other:__________________________(please specify) ___ Yes ___ No

c. Other:__________________________(please specify) ___ Yes ___ No

d. Other:__________________________(please specify) ___ Yes ___ No

e. Other :__________________________(please specify) ___ Yes ___ No

f. Other:__________________________(please specify) ___ Yes ___ No

15. How often do you have back pain and/or back stiffness when getting out of bed in the morning?
_____Never
_____Seldom
_____Often
_____Always

16. How often do you get back pain and/or back stiffness when driving or riding in a car within 1 hour?
_____Never

_____Seldom
_____Often
_____Always

17. What is the most you weighed in your life, excluding pregnancy? _______ lbs.

18. What was your weight when you were 20 years old? ________ lbs.

19. Marital Status:
____ Never married (Single)
____ Married
____ Divorced
____ Separated
____ Widowed/Widowered

20. What is the highest grade in school that you completed?
____ 8th grade or less
____ Some high school
____ High school graduate or GED
____ Some college
____ College graduate (Bachelor's Degree or higher)

21. Have you ever smoked tobacco?
___ Never
___ Yes, current
___ Yes, but smoked in the past
If never, go to question 22…otherwise
How old were you when you started smoking? _____ *years old*
How old were you when you quit smoking?_____ *years old*
On average, how many cigarettes did you smoke per day?______ *Cigarettes per day*

22. In the past, have you ever had a problem with alcohol? ____ Yes ____ No
    a. If yes, approximately how many years ago? _________ Years

23. What is your race/ethnicity is (if you wish to select more than one please either contact a research assistant or select "Other and type in your choices):
____ Caucasian or White
____ Hispanic or Latino
____ African American or Black
____ Asian
____ Pacific Islander or Native Hawaiian
____ Native American or Alaskan Native
____ Other (please specify)___________________________________
____ Decline to answer this question.

24. For women only:

Are you currently:
a. Pregnant? ____ Yes ____ No
    i. If yes, when is your due date ________/_______/_______
    Month Day Year

b. How many times have you been pregnant? _______

c. How many children have you given birth to? ______

25. How many brothers do you have (biological)? ________

26. How many sisters do you have (biological)? ________

27. Has any one in your family (blood relatives only) ever had a pinched nerve, Sciatica, or Lumbar Radiculopathy in the lower back? _____ Yes _____ No

    a. If yes, write in how many of each

Biological Sister(s)		Biological Brother(s)	
Biological Mother		Biological Father	

28. Has any one in your family (blood relatives only) ever been diagnosed Cervical Radiculopathy or a pinched nerve in the neck? _____ Yes _____ No

    a. If yes, write in how many of each

Biological Sister(s)		Biological Brother(s)	
Biological Mother		Biological Father	

29. How often do you have family problems that irritate or bother you?
____ Never
____ Sometimes
____ Often
____ Always

30. How often during the past month have you felt uneasy?

____ Never
____ Sometimes
____ Often
____ Always

31. How well do you sleep at night?
____ Very well
____ Well
____ Fair
____ Poorly
____ Very poorly

32. How often during the past month have you felt “on the edge”?
____ Never
____ Sometimes
____ Often
____ Always

33. How often during the past month have you felt tense?
____ Never
____ Sometimes
____ Often
____ Always

34. How often during the past month has your sleep been restless?
____ Never
____ Sometimes
____ Often
____ Always

35. How often are you <u>physically</u> exhausted after work?
____ Never
____ Sometimes
____ Often
____ Always

36. How often are you <u>mentally</u> exhausted after work?
____ Never
____ Sometimes
____ Often
____ Always

37. How often during the past month have you felt “down”, blue or depressed?
____ Never
____ Sometimes

____ Often
____ Always

38. How often during the past month have you felt nervous or anxious?
____ Never
____ Sometimes
____ Often
____ Always

The next group of questions pertain to feelings you have concerning your job.

39. All in all, how satisfied are you with your job?
____ Very satisfied
____ Somewhat Satisfied
____ A little satisfied
____ Not at all satisfied

40. My job requires working very fast.
____ Strongly agree
____ Agree
____ Disagree
____ Strongly Disagree

41. My job requires working very hard (physically).
____ Strongly agree
____ Agree
____ Disagree
____ Strongly Disagree

42. I am NOT asked to do an excessive amount of work.
____ Strongly agree
____ Agree
____ Disagree
____ Strongly Disagree

43. I have enough time to get the job done.
____ Strongly agree
____ Agree
____ Disagree
____ Strongly Disagree

44. I am free from conflicting demands that others make.

____ Strongly agree
____ Agree
____ Disagree
____ Strongly Disagree

45. How much influence or control do you have over the variety of tasks you perform?
____ Very Much
____ Much
____ Moderate Amount
____ A Little
____ Very Little

46. How much influence or control do you have over the order in which you perform tasks at work?
____ Very Much
____ Much
____ Moderate Amount
____ A Little
____ Very Little

47. How much influence or control do you have over the pace of your work, that is how fast or slow do you work?
____ Very Much
____ Much
____ Moderate Amount
____ A Little
____ Very Little

48. How much influence or control do you have over the extent to which you can work ahead and take a short break during work hours?
____ Very Much
____ Much
____ Moderate Amount
____ A Little
____ Very Little

49. How firm or hard is the mattress you sleep on?
____ Hard
____ Medium
____ Soft

50. On average, about how many total hours of sleep do you get per night (don't count time you are laying awake or trying to fall asleep)?

I average _______ total hours of sleep per night

51. Please look at the sleep positions below. Choose and rank up to two that are closest to the positions you think you use at night. If A, B, or C is selected, please indicate which side you sleep on.
______1st most common ______right side ______left side _____ both equally
_____ 2nd most common ______right side ______left side _____ both equally

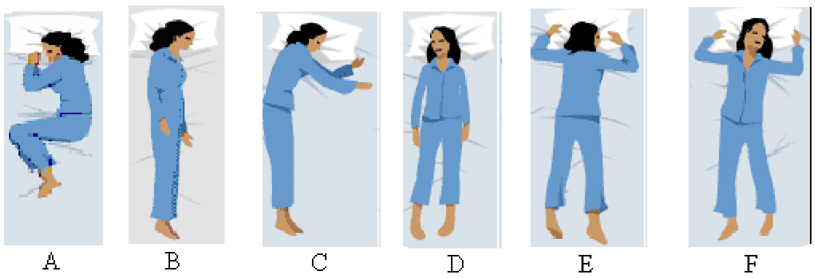

Modified Work APGAR

Please place a checkmark in the box corresponding with how you feel about each of the following statements.

		Almost always	Some of the time	Hardly ever
52.	I am satisfied that I can turn to a fellow worker for help if something is troubling me.			
53.	I am satisfied with the way my fellow workers talk things over with me and share problems with me.			
54.	I am satisfied that my fellow workers accept and support my new ideas and thoughts.			
55.	I am satisfied with the way my fellow workers respond to my emotions, such as anger, sorrow, or laughter.			
56.	I am satisfied with the way my fellow workers and I share time together.			
57.	I enjoy the tasks involved in my job.			

58.	I am satisfied with how well I get along with my closest or immediate supervisor.			

Modified Zung Depression Index

Please indicate for each of the following questions which answer best describes how you have been feeling recently.				
	Rarely or none of the time (less than 1 day per week)	Some or little of the time (1–2 days per week)	A moderate amount of time (3–4 times per week)	Most of the time (5–7 days per week)
59. I feel downhearted and sad				
60. I feel that nobody cares				
61. I get tired for no reason				
62. I feel that I am useful and needed				
63. I am still able to enjoy those things I used to				
64. I eat as much as I used to				
65. I am more irritable than usual				
66. I feel hopeful about the future				

Thank you for completing the questionnaire.

A.2 Structured Interview

Plant: ______________________
Subject ID #____________________
Date: ______________________
Time:_______________________
Examiner 1:___________________
Examiner 2:___________________

Back Baseline Structured Interview (to be completed on a laptop computer)

59. Have you ever been told by a health care professional (medical doctor/chiropractor) that you have any of the following:

a. Rheumatoid arthritis, Lupus, or another inflammatory arthritis (not typical Osteoarthritis or Degenerative Arthritis). ___ Yes ___ No
Approximately how many years ago was this diagnosed? _________Years

b. Osteoarthritis or Degenerative Arthritis ___ Yes ___ No

i. If yes, what joints have been affected? (check all that apply)

☑	Body Part	Which side is affected?			Approximately how many years ago was this diagnosed?
		Right	Left	Both	
☐	Fingers				Years
☐	Wrists				Years
☐	Elbows				Years
☐	Shoulders				Years
☐	Neck				Years
☐	Upper Back				Years
☐	Middle Back				Years
☐	Low Back				Years
☐	Hips	☐	☐	☐	Years
☐	Knees	☐	☐	☐	Years
☐	Ankles	☐	☐	☐	Years
☐	Toes	☐	☐	☐	Years

c. Fibromyalgia? ___ Yes ___ No

d. Myofascial Pain Syndrome? ___ Yes ___ No

e. Infection in the back bone (Osteomyelitis). ___ Yes ___ No
What bone(s) were infected:___________________________(please specify)

f. Osteoporosis:___________________________(please specify) ___ Yes ___ No
Please specify what bones were osteoporotic______________________________

g. Have you ever had a broken bone or fracture? ___ Yes ___ No

If yes, which bone(s) did you fracture?

**Body Part**	Check if yes	Right	Left	Bilateral
Foot / Ankle				
Calf / Tibia / Fibula (Lower Leg)				
Thigh / Femur				
Coccyx (Tail Bone)				
Pelvis				
Low Back / Lumbar Spine				
Middle Back / Thoracic Spine				
Neck / Cervical Spine				
Head / Skull				
Sternum (Breast Bone)				
Rib (s)				
Collar Bone / Clavicle				
Upper Arm / Humerus				
Lower Arm / Radius / Ulna				
Wrist				
Finger(s)				

60. Have you **EVER** had an accident or sudden injury to your Neck, Middle, or Lower Back (such as a motor vehicle accident, whiplash, sports injury, fracture, slip, trip, or fall)?
___Yes ___ No

a. If yes, please give year(s) of each:

Body Part	Approx. Year(s)	Did it occur at work?	Cause (check all that apply)
Neck		☐ Yes ☐ No ☐ Unsure	☐ MVA ☐ Sports ☐ Slip, Trip, Fall ☐ Unsure ☐ Other________________________________
Middle Back		☐ Yes ☐ No ☐ Unsure	☐ MVA ☐ Sports ☐ Slip, Trip, Fall ☐ Unsure ☐ Other________________________________
Lower Back		☐ Yes ☐ No ☐ Unsure	☐ MVA ☐ Sports ☐ Slip, Trip, Fall ☐ Unsure ☐ Other________________________________

61. Have you ever had surgery on your neck, back, legs, or feet? _____ Yes _____No
    a. If yes, in what body part did you have surgery on?

	**Surgery**		**Describe**
Type of Surgery	Yes	No	
Neck			☐ Disk Removal ☐ Vertebral Fusion ☐ Other (specify)
Middle Back			☐ Disk Removal ☐ Vertebral Fusion ☐ Other (specify)
Low Back			☐ Disk Removal ☐ Vertebral Fusion ☐ Laminectomy ☐ Other (specify)
Hip			☐ Hip Replacement ☐ Other (specify)
Knee			☐ Cartilage Removed ☐ Ligament Repair ☐ Knee Replacement ☐ Other (specify)
Foot			☐ Bunions / Corns / Calluses ☐ Other (specify)

62. Have you **ever** been told by a health care professional (physican, chiropractor, etc.) that you have any of the following:

Disorder	Yes	No		Right	Left	Unsure
Neck Strain, Neck Sprain						
Pinched Nerve in the Neck (Cervical Radiculopathy)						
Bulging Disk in the Neck						
Degenerative Spine/Disk disease in the neck (Cervical)						
Lower Back Pain, Lower Back Strain, Lower Back Sprain						
Pinched nerve in the lower back (Sciatica)						
Sacroiliac Joint Dysfunction, Sacroiliitis						
Bulging Disk in the Lower Back						
Degenerative Spine/Disk disease in the low back (Lumbar)						
Spondylolisthesis						
Spina bifida						

Scoliosis						
Problem with Lower Spine / Vertebral Bone Alignment						
Other						
Other						
Other						

The following questions ask about your history of LOW BACK PAIN in your life.

63. Have you EVER had pain in your **Low Back Pain** that **lasted at least 7 days**?

______Yes ______No

If answer to question 5 is yes, skip to question 6.

a. Have you EVER had pain in your **Low Back Pain** that **lasted at least 3 days**?

______Yes ______No

If answer to question 5a is no, skip to question 11.

64. About how old were you when you first experienced **Low Back Pain** that lasted at least (3 or 7 depending on question 5) days?

______years old

65. How many episodes of **Low Back Pain** have you ever had lasting at least (3 or 7) days?

_______episodes

a. Approximately how many of those episodes (answer from #7) lasting at least (3 or 7) days do you estimate were work-related? _______episodes

b. How long ago was your **most recent** episode of **Low Back Pain** lasting at least (3 or 7) days?

____ Years _____ Months ______ Weeks ______ Days

c. How long did that **most recent** episode lasting at least (3 or 7) days last?

____ Years ____ Months

____Weeks

____Days

66. What was the worst **Low Back Pain** you ever experienced? (please refer to pain scale below)

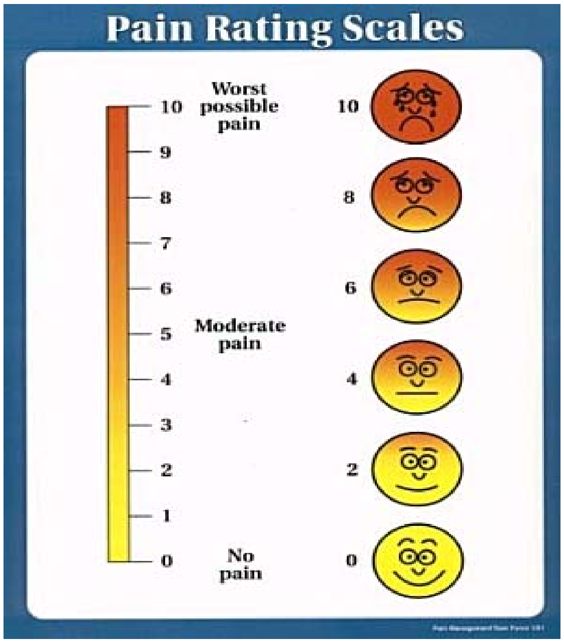

______/10

67. How long was the longest episode of **Low Back Pain** that you EVER experienced?
____ Months ____Weeks ____Days

68. Which of the following treatments or tests have you EVER had for **Low Back**? (check all that apply)

Treatment option	Yes	No	Approximate number of appointments or times
Medical Doctor (M.D.) or Doctor of Osteopathy (D.O.)			Appts.
Chiropractor			Appts.
Physical Therapy (P.T.)			Appts.
Massage Therapist			Appts.
Plain X-Ray			x-ray series
Magnetic Resonance Image (MRI)			MRI scans
Computed Tomography Imaging (CT Scan, CAT Scan)			CT scans
Over the Counter Medications (e.g. Tylenol, Aspirin, Advil, etc.)			Courses of medication
Prescription Non-Narcotic Medications (e.g. prescription of Ibuprophen/Motrin, Vioxx, Celebrex, etc.)			Rx
Prescription of Narcotic Medications (e.g. Lortab, Tylenol with Codeine, Vicodin, etc.)			Rx
Epidural Cortisone Injections			Injx

Work Hardening			Rx for WH
Back Belt / Back Braces			Back Belt / Braces
I don't recall			
No Treatment			

If "Yes" to question 5 then participants will not see questions 11.

69. Have you ever had **Low Back Pain** that lasted **at least 24 hours**? ____ Yes _____ No

    a. If yes, did you see a doctor (MD, DO, or Chiropractor) for these back pain(s)? ____ Yes _____ No

If answer to question 11 is "No", skip to question 15.

70. Not including car or other accidents, did you ever miss work because of **Low Back Pain** you thought was from work?
    ______Yes ______No

    a. If yes, approximately how many days have you missed for **Low Back Pain** altogether? ______ days

    b. What was the longest period of time you missed work because of **Low Back Pain**? ____ Months ____Weeks ____Days

    c. Did you report the **Low Back Pain** to your employer? ____ Yes _____ No

71. Not including car or other accidents, have you ever been on light / restricted / modified duty to **Low Back Pain**?
    ______Yes ______No

    a. If yes, approximately how many days have you been on light / restricted / modified duty for **Low Back Pain** altogether?
    ____ Months ____Weeks ____Days

    b. What was the longest period of time you were on light / restricted / modified duty for **Low Back Pain**?
    ____ Months ____Weeks ____Days

72. Not including car or other accidents have you ever changed jobs because of **Low Back Pain** other than temporary assignments?
    ______Yes ______No

<u>The following questions ask about your history of MIDDLE BACK PAIN in your life.</u>

73. Have you EVER had pain in your **<u>Middle Back Pain</u>** that **lasted at least 3 days**?
______Yes ______No
If answer to question 14 is no, skip to question 23.

74. About how old were you when you first experienced **<u>Middle Back Pain</u>** that lasted at least 3 days?
______years old

75. How many episodes of **<u>Middle Back Pain</u>** have you <u>ever</u> had lasting at least 3 days?
_______episodes

    a. Approximately how many of those (answer from #16) episodes lasted at least 7 days?
    _______episodes

    b. Approximately how many of those episodes (answer from #16 A) lasting at least 7 days do you estimate were work-related? _______episodes

76. What was the worst **<u>Middle Back Pain</u>** you ever experienced? (please refer to pain scale below)

______/10

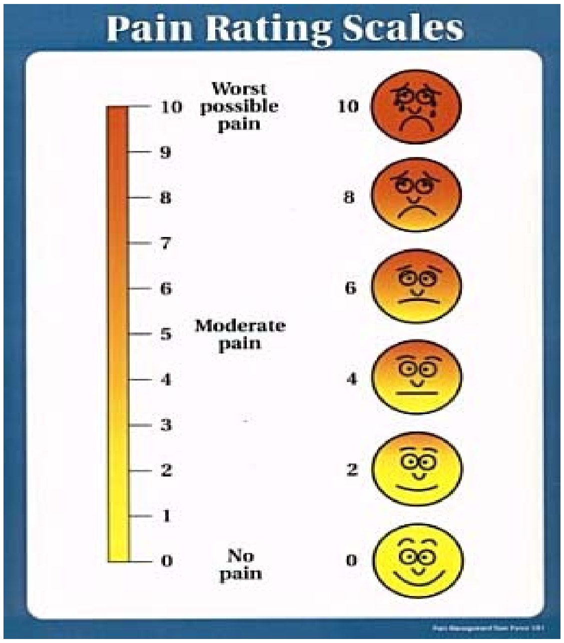

How long was the longest episode of **Middle Back Pain** that you EVER experienced?

____ Months ____Weeks ____Days

77. Which of the following treatments or tests have you EVER had for **Middle Back**? (check all that apply)

Treatment option	Yes	No	Approximate number of appointments or times
Medical Doctor (M.D.) or Doctor of Osteopathy (D.O.)			Appts.
Chiropractor			Appts.
Physical Therapy (P.T.)			Appts.
Massage Therapist			Appts.
Plain X-Ray			x-ray series
Magnetic Resonance Image (MRI)			MRI scans
Computed Tomography Imaging (CT Scan, CAT Scan)			CT scans
Over the Counter Medications (e.g. Tylenol, Aspirin, Advil, etc.)			Courses of medication
Prescription Non-Narcotic Medications (e.g. prescription of Ibuprophen/Motrin, Vioxx, Celebrex, etc.)			Rx
Prescription of Narcotic Medications (e.g. Lortab, Tylenol with Codeine, Vicodin, etc.)			Rx
Epidural Cortisone Injections			Injx
Work Hardening			Rx for WH
Back Belt / Back Braces			Back Belt / Braces
I don't recall			
No Treatment			

78. Not including car or other accidents, did you ever miss work because of **Middle Back Pain** you thought was from work?

______Yes ______No

a. If yes, approximately how many days have you missed for **Middle Back Pain** altogether?
______ days

b. What was the longest period of time you missed work because of **Middle Back Pain**?
____ Months ____Weeks ____Days

c. Did you report the **Middle Back Pain** to your employer?
____ Yes _____ No

79. Not including car or other accidents, have you ever been on light / restricted / modified duty to **Middle Back Pain**?

______Yes ______No

a. If yes, approximately how many days have you been on light / restricted / modified duty for **<u>Middle Back Pain</u>** altogether?

____ Months ____Weeks ____Days

b. What was the longest period of time you were on light / restricted / modified duty for **<u>Middle Back Pain</u>**?

____ Months ____Weeks ____Days

Not including car or other accidents have you ever changed jobs because of **<u>Middle Back Pain</u>** other than temporary assignments?

______Yes ______No

<u>The following questions ask about your history of NECK PAIN in your life.</u>

80. Have you EVER had pain in your **<u>Neck Pain</u>** that **lasted at least 3 days**?

______Yes ______No

If answer to question 23 is no, skip to question 32.

81. About how old were you when you first experienced **<u>Neck Pain</u>** that lasted at least 3 days?

______years old

82. How many episodes of **<u>Neck Pain</u>** have you <u>ever</u> had lasting at least 3 days?

_______episodes

a. Approximately how many of those (answer from #25) episodes lasted at least 7 days?
_______episodes

b. Approximately how many of those episodes (answer from #25 A) lasting at least 7 days do you estimate were work-related? _______episodes

c. How long ago was your **most recent** episode of **<u>Neck Pain</u>** lasting at least 7 days?
____ Years _____ Months ______ Weeks ______ Days

d. How long did that **most recent** episode lasting at least 7 days last?
____ Years ____ Months ____Weeks ____Days

83. What was the worst **<u>Neck Pain</u>** you ever experienced? (please refer to pain scale below)

______/10

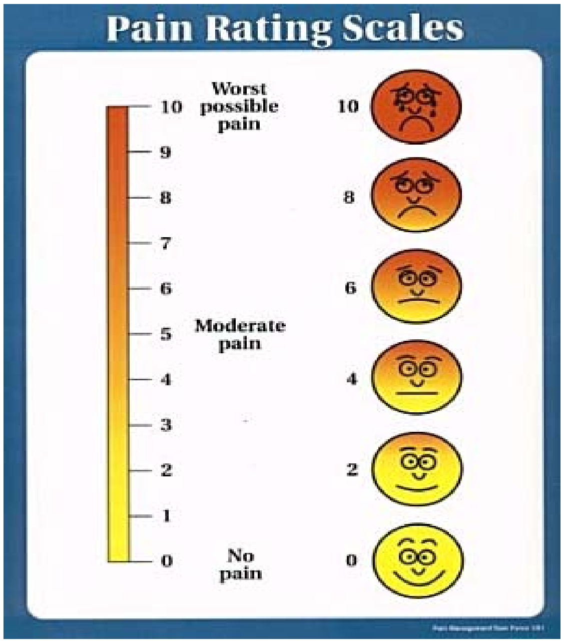

How long was the longest episode of **Neck Pain** that you EVER experienced?
____ Months ____Weeks ____Days

84. Which of the following treatments or tests have you EVER had for **Neck Pain**? (check all that apply)

Treatment option	Yes	No	Approximate number of appointments or times
Medical Doctor (M.D.) or Doctor of Osteopathy (D.O.)			Appts.
Chiropractor			Appts.
Physical Therapy (P.T.)			Appts.
Massage Therapist			Appts.
Plain X-Ray			x-ray series
Magnetic Resonance Image (MRI)			MRI scans
Computed Tomography Imaging (CT Scan, CAT Scan)			CT scans
Over the Counter Medications (e.g. Tylenol, Aspirin, Advil, etc.)			Courses of medication
Prescription Non-Narcotic Medications (e.g. prescription of Ibuprophen/Motrin, Vioxx, Celebrex, etc.)			Rx
Prescription of Narcotic Medications (e.g. Lortab, Tylenol with Codeine, Vicodin, etc.)			Rx

Epidural Cortisone Injections			Injx
Work Hardening			Rx for WH
Back Belt / Back Braces			Back Belt / Braces
I don't recall			
No Treatment			

85. Not including car or other accidents, did you ever miss work because of **Neck Pain** you thought was from work?

    ______Yes ______No

    a. If yes, approximately how many days have you missed for **Neck Pain** altogether?
    ______ days

    b. What was the longest period of time you missed work because of **Neck Pain**?
    ____ Months ____Weeks ____Days

    c. Did you report the **Neck Pain** to your employer?
    ____ Yes _____ No

86. Not including car or other accidents, have you ever been on light / restricted / modified duty to **Neck Pain**?

    ______Yes ______No

    a. If yes, approximately how many days have you been on light / restricted / modified duty for **Neck Pain** altogether?
    ____ Months ____Weeks ____Days

    b. What was the longest period of time you were on light / restricted / modified duty for **Neck Pain**?
    ____ Months ____Weeks ____Days

87. Not including car or other accidents have you ever changed jobs because of **Neck Pain** other than temporary assignments?______Yes ______No

88. PAIN ASSESSMENT

At the current time or at any time in the **past month** have you had any pain, ache, and/or burning, in any of the following body parts? (check all that apply and refer to the body diagram so the worker can note <u>all</u> areas that apply).

**Body part**	Yes	No	Pain / Ache / Burning in this body part	Average Pain Severity Rating (0-10)	Current (today) Pain / Ache / Burning Symptoms	Total Pain Duration (could be >30 days… e.g. 365 for 1 year)
Neck **(A)**					□Yes □No	Days
Shoulder(s)						
Interscapular **(F)**			□Yes □No		□Yes □No	Days
R. Nape of the Neck **(C)**			□Yes □No		□Yes □No	Days
R. Periscapular **(G)**			□Yes □No		□Yes □No	Days
R. Shoulder (Glenohumeral) **(H)**			□Yes □No		□Yes □No	Days
R. Upper Arm **(K)**			□Yes □No		□Yes □No	Days
L. Nape of the Neck **(B)**			□Yes □No		□Yes □No	Days
L. Periscapular **(E)**			□Yes □No		□Yes □No	Days
L. Shoulder (Glenohumeral) **(D)**			□Yes □No		□Yes □No	Days
L. Upper Arm **(I)**			□Yes □No		□Yes □No	Days
Middle Back **(J)**			□Yes □No		□Yes □No	Days
Lower Back						
Lumbar Immediately Paraspinal **(M)**			□Yes □No		□Yes □No	Days
R. Lumbar **(N)**			□Yes □No		□Yes □No	Days
L. Lumber **(L)**			□Yes □No		□Yes □No	Days
R. Gluteal **(P)**			□Yes □No		□Yes □No	Days
L. Gluteal **(O)**			□Yes □No		□Yes □No	Days
Legs						
R. Thigh **(R)**			□Yes □No		□Yes □No	Days
L. Thigh **(Q)**			□Yes □No		□Yes □No	Days
R. Upper Calf **(T)**			□Yes □No		□Yes □No	Days

Need to fill out pain diagram

L. Upper Calf **(S)**			□Yes □No		□Yes □No	Days
R. Lower Calf/foot **(V)**			□Yes □No		□Yes □No	Days
L. Lower Calf/foot **(U)**			□Yes □No		□Yes □No	Days

a. If yes, which of the above, if any, were from an accident (e.g. a car accident, slip, trip, fall, etc.)?

89. Have you ever had pain in the lower back or buttock that traveled down a leg into a calf? ____ Yes ____ No _____ Right _____ Left _____ Both at the same time ____ Both legs at different times

a. If yes, at it's longest, approximately how long did this type of pain last (mark only if pain in that leg)?
Right: ___ Days ___ Weeks ___ Months ___ Years **Left:** ___ Days ___ Weeks ___ Months ___ Years

90. Did you have numbness and/or tingling in either of your legs **in the past month**? Yes _____ No _____

a. If yes, please mark where in the table below (mark all that apply).

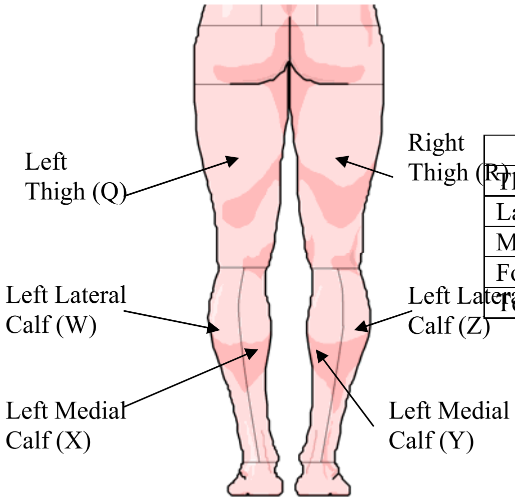

	Yes	No	Left	Right
Thigh (Q & R)				
Lateral Calf (W & Z)				
Medial Calf (X & Y)				
Foot				
Toes				

91. Did the tingling and/or numbness occur at the same time as low back pain? Yes _____ No _____

92. Did the tingling and/or numbness occur only with sitting on a leg and causing it to "fall asleep"?
Yes _____ No _____

93. Have you ever received workers compensation for time off work for a work related injury, whether for the back or any other part of the body? ____ Yes ____ No
    a. If yes, was it for a back related injury? ______ Yes _____ No

A.3. Physical Examination

Structured Interview Low Back Pain Diagram #1

Typical Low Back Pain in the past month

This is to be completed by anyone indicating pain in Lumbar Immediately Paraspinal **(M),** Right Lumbar **(N)** Left Lumber **(L),** Right Gluteal **(P),** and/or Left Gluteal **(O)**

94. **Circle** the area of the low back where you typically experienced pain **in the past month**.

95. **Mark with an "X"** on the diagram where you experienced the **WORST** pain.

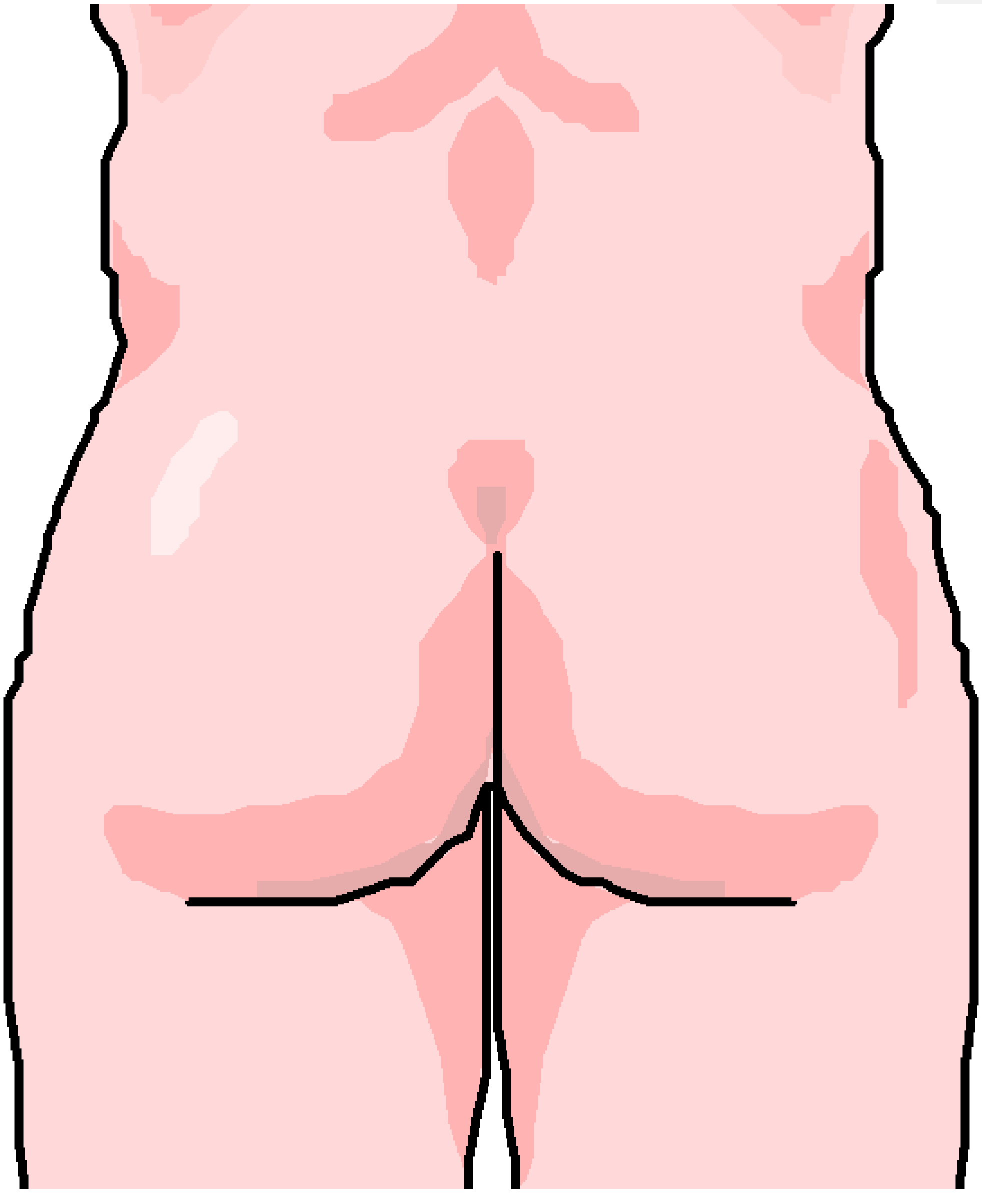

Plant: ___________________________
Subject ID #______________________
Date: ___________________________

Back Vital Statistics Data Collection

1. Heart Rate _________beats/min

2. Blood Pressure ________mmHg / ________mmHg
   Systolic Diastolic

Test	Yes	No
R. Foot Only Toe Raise 10 Times (able to complete task?)		
L. Foot Only Toe Raise 10 Times (able to complete task?)		
Heel Walk 5 Paces on each leg (able to complete task?)		
Toe Walk 5 Paces on each leg (able to complete task?)		

3. Gait (10 paces) __ Normal __Antalgic
   __ Other describe:______________________________

4. Chest Circumference ______ cm

5. Waist Circumference ______ cm

6. Hip Circumference ______ cm

7. Measured Middle Finger fingertip to floor distance (knees locked, without shoes)

Finger	Neutral Upright	Lateral Flexion	Forward Spinal Flexion
Right (cm)			
Left (cm)			

8. Goniometer measurements (landmarks are greater Trochanter to the posterior acromion process and middle knee joint)
   a. Neutral _______degrees
   b. Full Flexion _______degrees
   c. Full Extension _______degrees

9. Measured Weight (Without Shoes) ____.__ kg

10. Measured Height (Without Shoes) ____.__ cm

11. Shoulder Width (Lateral Posterior Acromion process tip to Lateral P. Acromion process) ______ cm

12. Hip to Shoulder length (Lateral Iliac crest to Posterior Acromion Process) ______ cm

13. Upper Arm length (Posterior Acromion Process to Upper Olecranon Process) ______ cm

14. Lower Arm length (Upper Olecranon Process to distal 3rd MCP joint) ______ cm

15. Horizontal Arm Length (Horizontal From Posterior Acromion to distal 3rd MCP Joint) ______ cm

16. Elbow Height (Upper Olecranon Process to floor, **with shoes**) ______ cm

17. Knee Height (Upper patella to floor, **with shoes**) ______ cm

Plant: ____________________
Subject ID #____________________
Date: ____________________

Back Physical Examination Form (all physicals)

Body region	Sign	1st Examiner + Present	1st Examiner - Absent	2nd Examiner + Present	2nd Examiner - Absent
Neck (seated)	R. Spurlings (Right Neck Rotation)				
	L. Spurlings (Left Neck Rotation)				
	Abnormal Cervical ROM (Flexion less than 2 cm from the sternum)				
	Evidence of Neck Surgery / Scar (Anterior or Posterior)				
	Superficial Neck Tenderness				
	General Overreaction to Examination				
Neck (seated)	Simulated Axial Loading				
Back (seated)	Simulated Axial Loading				

Lumbar Back	**Seated Physical Exam Maneuver**	+ Present	- Absent	+ Present	- Absent
	R. "+" **Seated** Straight Leg Raise (Leg Pain below the knee not hamstring tightness, 60º or less)				
	L. **"+" Seated** Straight Leg Raise (Leg Pain below the knee not hamstring tightness, 60º or less)				

**Seated Test**	1st Examiner	2nd Examiner
R. Patellar Tendon Reflex Grade (0 - 4+)		
L. Patellar Tendon Reflex Grade (0 - 4+)		
R. Achilles Tendon Reflex Grade (0 – 4+)		
L. Achilles Tendon Reflex Grade (0 – 4+)		
R. Extensor Hallicus Muscle Strength Grade (1-5 / 5)	/ 5	/ 5
L. Extensor Hallicus Muscle Strength Grade (1-5 / 5)	/ 5	/ 5

Lumbar Back	**Physical Exam Maneuver**	+ Present	- Absent	+ Present	- Absent
(recumbent)	R. **"+" Recumbent** Straight Leg Raise (Leg Pain, ≤ 60º)				
	L. "+" **Recumbent** Straight Leg Raise (Leg Pain, ≤ 60º)				
	R. Sacroiliac Joint Stress Test				
	L. Sacroiliac Joint Stress Test				

(standing)	Simulated Rotation (20 to 30 degrees)				
	General Overreaction to Low Back Examination				
	Superficial Low Back Tenderness				
	Evidence of back surgery / scar				

Neck Pain Tender Point Evaluation

Directions: Using 4 kg of force, evaluate all tender points, checking all points where "**pain**" is indicated. **Circle the <u>MOST PAINFUL</u>** of all tender points in the neck (if any).

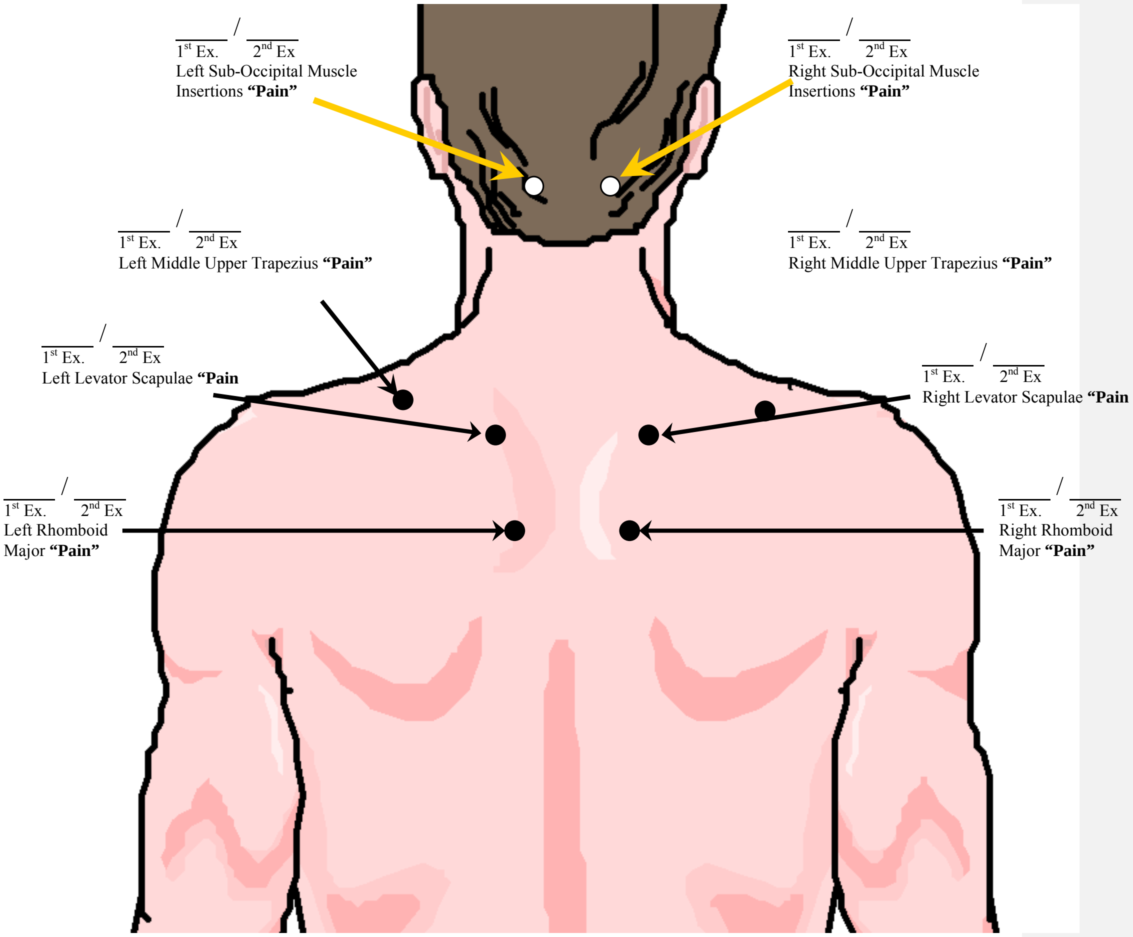

Directions: Using 4 kg of force, evaluate all tender points, checking all points where **"pain"** is indicated. **Circle the MOST PAINFUL** of all tender points in the Low Back (if any).

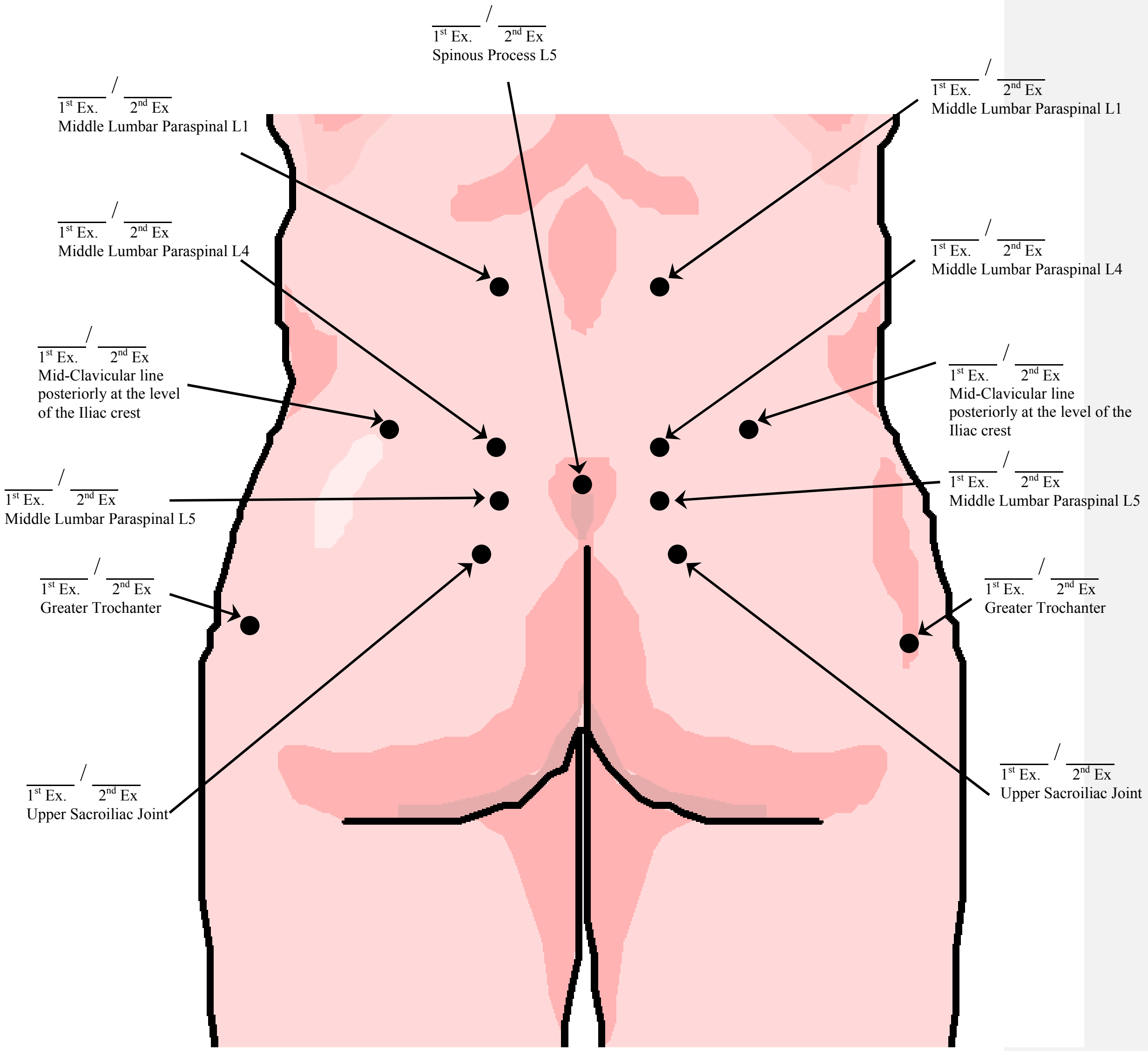

18. Signs of Rheumatoid Arthritis ____ Yes ____ No

19. Heberden's Nodes ____ Yes ____ No
If yes, which joint(s)___________________________________

20. Bouchard's Nodes ____ Yes ____ No
If yes, which joint(s)___________________________________

Other findings in the physical exam (e.g. Sensory Exam If Indicated):

Body Part	Test performed	Positive finding	Negative finding	Examiner	
				1st	2nd

Overall Diagnostic Impression

(check appropriate boxes)

		Yes	No
Past history of **Right** sciatica	Right		
Past history of **Left** sciatica			
Current history of **Right** sciatica (**L5** Nerve Root)	Right		
Current history of **Left** sciatica (**L5** Nerve Root)			
Current history of **Right** sciatica (**S1** Nerve Root)	Right		
Current history of **Left** sciatica (**S1** Nerve Root)			
Past history of Low Back Pain lasting at least 7 days			
Current history of Low Back Pain lasting at least 7 days			
Other (describe)			
Other (describe)			

A.4. Monthly Follow-up

Gender: ______________________
Date: ______________________
Last Visit Date :________________
Days Since Last Visit:___________
Time:______________________
Plant:______________________
Subject ID:__________________

Low Back Pain Monthly Questionnaire (to be completed on a laptop computer)

1. Absent ___ Yes ___ No

If Yes, Why is the participant absent? (Please circle all that apply).
Ill / sick, Vacation, FMLA, Surgery / Medical Appt, Laid off, Terminated, Quit,
Declined Participation, Declined participation (this month only), OTHER….

If quit, terminated, or laid off, when was their last day here?

____
MM / DD / YYYY

2. **Last time we were here you had pain in**:
All areas of pain from previous month will be checked and previous pain rating present.

☑	Body Part	Previous Pain Rating	Did it go away?	Is it the same?	Present Pain Rating?	Percent of days you have pain?	If the pain stopped how many days ago did it stop?
☐	Lumbar Immediately Paraspinal (M)		Yes /No	Yes /No			
☐	R. Lumbar (N)		Yes /No	Yes /No			
☐	L. Lumbar (L)		Yes /No	Yes /No			
☐	R. Gluteal (P)		Yes /No	Yes /No			
☐	L. Gluteal (O)		Yes /No	Yes /No			
☐	R. Thigh (R)		Yes /No	Yes /No			
☐	L. Thigh (Q)		Yes /No	Yes /No			

☐	R. Upper Calf (T)		Yes /No	Yes /No			
☐	L. Upper Calf (S)		Yes /No	Yes /No			
☐	R. Lower Calf/foot (V)		Yes /No	Yes /No			
☐	L. Lower Calf/foot (U)		Yes /No	Yes /No			

3. **Last time we were here you had numbness and tingling in:**
   Previous percent of days will be filled in.

☑	**Body Part**	**Previous % of days**	**Did it go away?**	**Is it the same?**	**Precent of days you have N/T?**	**Does the N/T occur at the same time as the low back pain?**	
☐	R. Thigh (R)		Yes / No			Yes / No	
☐	R. Lateral Calf (Z)		Yes / No			Yes / No	
☐	R. Medial Calf (Y)		Yes / No			Yes / No	
☐	R. Foot (S)		Yes / No			Yes / No	
☐	R. Toes (U)		Yes / No			Yes / No	
☐	L. Thigh (Q)		Yes / No			Yes / No	
☐	L. Lateral Calf (W)		Yes / No			Yes / No	
☐	L. Medial Calf (X)		Yes / No			Yes / No	
☐	L. Foot (T)		Yes / No			Yes / No	
☐	L. Toes (V)		Yes / No			Yes / No	

4. Do you have any <u>NEW PAIN</u> since your last follow-up ___ days ago in your

**Body part**	Pain / Ache / Burning in this body part	Average Pain Severity Rating (0-10)	Current (today) Pain / Ache / Burning Symptoms	Total Pain Duration
Lumbar Immediately Paraspinal **(M)**	□Yes □No		□Yes □No	Days
R. Lumbar **(N)**	□Yes □No		□Yes □No	Days
L. Lumber **(L)**	□Yes □No		□Yes □No	Days

R. Gluteal **(P)**	□Yes □No		□Yes □No	Days
L. Gluteal **(O)**	□Yes □No		□Yes □No	Days
Legs				
R. Thigh **(R)**	□Yes □No		□Yes □No	Days
L. Thigh **(Q)**	□Yes □No		□Yes □No	Days
R. Upper Calf **(T)**	□Yes □No		□Yes □No	Days
L. Upper Calf **(S)**	□Yes □No		□Yes □No	Days
R. Lower Calf/foot **(V)**	□Yes □No		□Yes □No	Days
L. Lower Calf/foot **(U)**	□Yes □No		□Yes □No	Days

PLEASE FILL OUT PAIN DIAGRAM FOR YOUR NEW LOW BACK

5. What do you think caused this New Pain?
   ______Unsure
   ______ Accident outside of work (slip, trip, fall, MVA, etc.)
   ______ Something outside of work (NOT an accident)
   ______ Accident at work (slip, trip, fall, MVA, etc.)
   ______ Something at work (Not an accident)
   ______ Relapse or aggrivation of previously reported pain

6. When did the Pain occur?
   (Don't answer if cause =”Unsure”
   ______Sudden (within 30 minutes)
   ______Same dau (after 30 mimutes)
   ______Next day or after
   ______Don't Know

7. Did a job evaluation team study the part of your job related to when/where you were injured?
   _______Yes
   _______No
   _______Not Applicable

8. Do you have any new NUMBNESS/TINGLING in your:

☑	**Body Part**	**Number of days you have had N/T.**	**How often do you have N/T upon awakening in the morning?**	**How often do you have N/T at night?**
☐	R. Thigh (R)			

☐	R. Lateral Calf (Z)			
☐	R. Medial Calf (Y)			
☐	R. Foot (S)			
☐	R. Toes (U)			
☐	L. Thigh (Q)			
☐	L. Lateral Calf (W)			
☐	L. Medial Calf (X)			
☐	L. Foot (T)			
☐	L. Toes (V)			

9. What do you think caused this NUMBNESS/TINGLING?
   ______Unsure
   ______ Accident outside of work (slip, trip, fall, MVA, etc.)
   ______ Something outside of work (NOT an accident)
   ______ Accident at work (slip, trip, fall, MVA, etc.)
   ______ Something at work (Not an accident)
   ______ Relapse or aggrivation of previously reported pain

10. When did the Numbness/Tingling occur?
   (Don't answer if cause ="Unsure"
   ______Sudden (within 30 minutes)
   ______Same dau (after 30 mimutes)
   ______Next day or after
   ______Don't Know

11. Does the N/T occur at the same time as the low back pain?
   ______Yes
   ______No

12. Since your follow-up ____ days ago, have you been on light duty because of Low Back Pain?
   ______Yes
   ______No

If yes, How many days ago or what day did you begin light duty?

_______________________ ___________

month day year days ago

When did you end or will you end working light duty?

____

month day year

13. Since your last follow-up ____ days ago, have you had any lost duty days due to Low Back Pain?
    ______Yes
    ______No

    If Yes, How many lost duty days did you have?
    ______days

14. Are you taking any medication or getting any treatment for your pain or numbness/tingling?
    ______Yes
    ______No

If YES, What types of treatment are you using?

☑	**Mid-Back**	Yes / No
☐	Back Surgery	
☐	Injection in the back	
☐	Back Brace	
☐	Chiropractor	
☐	Physician	
☐	PT or OT	
☑	**Low Back**	Yes / No
☐	Back Surgery	Yes / No
☐	Injection in the back	Yes / No
☐	Back Brace	Yes / No

☐	Chiropractor	Yes / No
☐	Physician	Yes / No
☐	PT or OT	Yes / No
	**Other**	
☐	NSAID or OTC Meds	Yes / No
☐	Prescription Meds not NSAID or Narcotic	Yes / No
☐	Prescription, Narcotic / painkillers	Yes / No
☐	Light Duty	Yes / No
☐	Aerobic Exercise	Yes / No
☐	Strength Exercise	Yes / No
☐	Stretching	Yes / No
☐	Other	Yes / No
☐	Other	Yes / No

15. Since we saw you _____ days ago, Have you:

Changed jobs / lines	Yes/No
Changed the number of hours you work	Yes / No
Started using new / different equipment	Yes / No
Changed your rotation schedule	Yes / No
Dhanged your production rate	Yes / No
Other	Yes / No

16. Does the participant complain of a "tired back" even though there is no new or recurrent Low Back Pain?
______Yes
______No

17. If you were absent at our last follow-up visit, what day did you return to work?

____
Month Day Year

18. If you were not here the last time we collected monthly follow-up data, why were you absent?
______ill
______Vacation
______Pregnancy
______Light Duty
______Injury
______Surgery or medical appt.
______Other

Monthly Follow-Up Low Back Pain Diagram #1

Typical Low Back Pain in the past month

This is to be completed by anyone indicating pain in Lumbar Immediately Paraspinal **(M),** Right Lumbar **(N)** Left Lumber **(L),** Right Gluteal **(P),** and/or Left Gluteal **(O)**

96. **Circle** the area of the low back where you typically experienced pain **in the past month**.

97. **Mark with an "X"** on the diagram where you experienced the **WORST** pain.

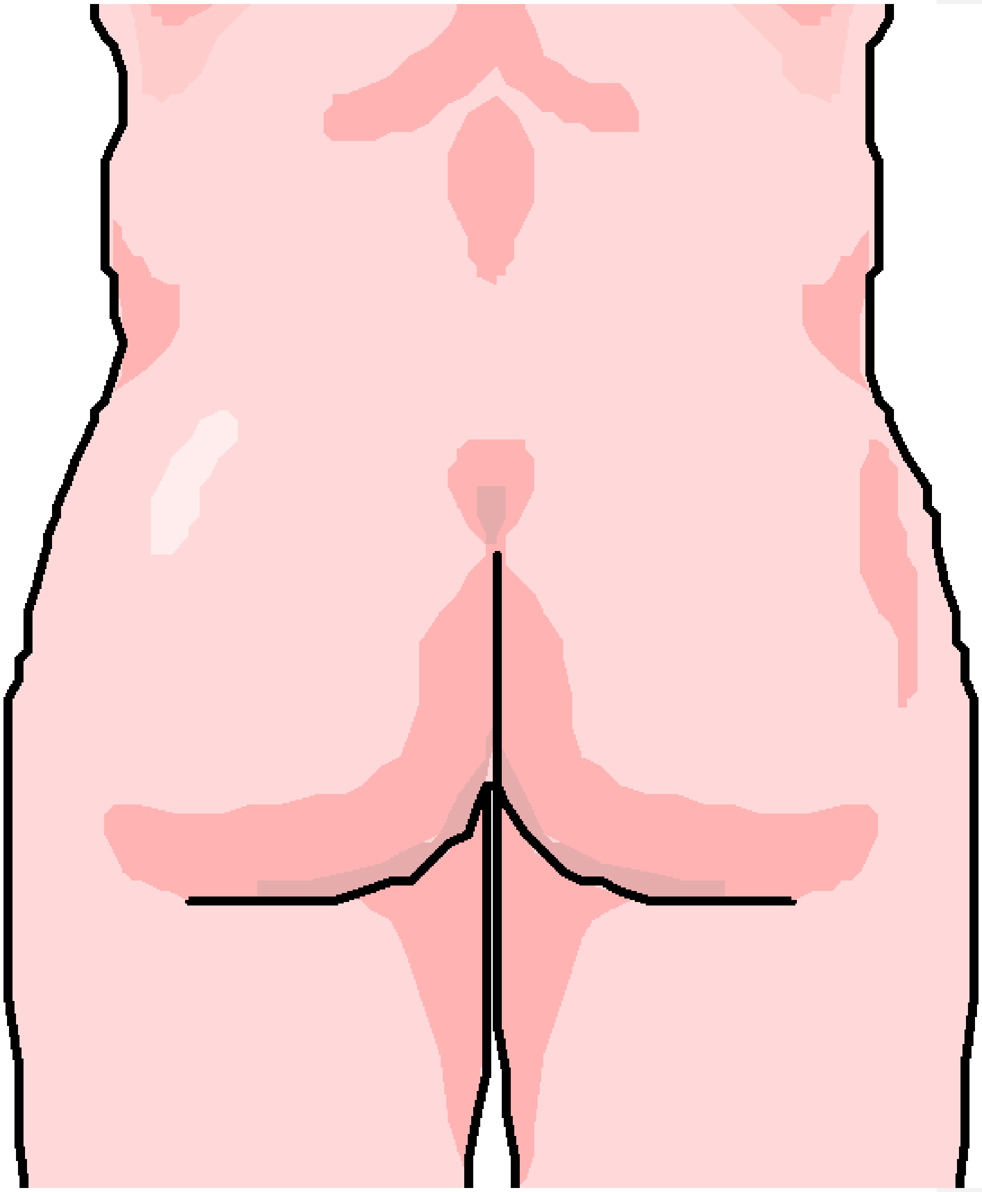

Low Back Pain Monthly Questionnaire (to be completed on a laptop computer)

19. Absent ___ Yes ___ No

If Yes, Why is the participant absent? (Please circle all that apply).
Ill / sick, Vacation, FMLA, Surgery / Medical Appt, Laid off, Terminated, Quit,
Declined Participation, Declined participation (this month only), OTHER….

If quit, terminated, or laid off, when was their last day here?

____
MM / DD / YYYY

20. **Last time we were here you had pain in**:
All areas of pain from previous month will be checked and previous pain rating present.

☑	Body Part	Previous Pain Rating	Did it go away?	Is it the same?	Present Pain Rating?	Percent of days you have pain?	If the pain stopped how many days ago did it stop?
☐	Lumbar Immediately Paraspinal (M)		Yes /No	Yes /No			
☐	R. Lumbar (N)		Yes /No	Yes /No			
☐	L. Lumbar (L)		Yes /No	Yes /No			
☐	R. Gluteal (P)		Yes /No	Yes /No			
☐	L. Gluteal (O)		Yes /No	Yes /No			
☐	R. Thigh (R)		Yes /No	Yes /No			
☐	L. Thigh (Q)		Yes /No	Yes /No			
☐	R. Upper Calf (T)		Yes /No	Yes /No			
☐	L. Upper Calf		Yes /No	Yes /No			

	(S)						
☐	R. Lower Calf/foot (V)		Yes /No	Yes /No			
☐	L. Lower Calf/foot (U)		Yes /No	Yes /No			

21. **Last time we were here you had numbness and tingling in:**
Previous percent of days will be filled in.

☑	**Body Part**	**Previous % of days**	**Did it go away?**	**Is it the same?**	**Precent of days you have N/T?**	**Does the N/T occur at the same time as the low back pain?**	
☐	R. Thigh (R)		Yes / No			Yes / No	
☐	R. Lateral Calf (Z)		Yes / No			Yes / No	
☐	R. Medial Calf (Y)		Yes / No			Yes / No	
☐	R. Foot (S)		Yes / No			Yes / No	
☐	R. Toes (U)		Yes / No			Yes / No	
☐	L. Thigh (Q)		Yes / No			Yes / No	
☐	L. Lateral Calf (W)		Yes / No			Yes / No	
☐	L. Medial Calf (X)		Yes / No			Yes / No	
☐	L. Foot (T)		Yes / No			Yes / No	
☐	L. Toes (V)		Yes / No			Yes / No	

22. Do you have any NEW PAIN since your last follow-up ___ days ago in your

**Body part**	Pain / Ache / Burning in this body part	Average Pain Severity Rating (0-10)	Current (today) Pain / Ache / Burning Symptoms	Total Pain Duration
Lumbar Immediately Paraspinal **(M)**	□Yes □No		□Yes □No	Days
R. Lumbar **(N)**	□Yes □No		□Yes □No	Days
L. Lumber **(L)**	□Yes □No		□Yes □No	Days
R. Gluteal **(P)**	□Yes □No		□Yes □No	Days
L. Gluteal **(O)**	□Yes □No		□Yes □No	Days
Legs				

R. Thigh **(R)**	□Yes □No		□Yes □No		Days
L. Thigh **(Q)**	□Yes □No		□Yes □No		Days
R. Upper Calf **(T)**	□Yes □No		□Yes □No		Days
L. Upper Calf **(S)**	□Yes □No		□Yes □No		Days
R. Lower Calf/foot **(V)**	□Yes □No		□Yes □No		Days
L. Lower Calf/foot **(U)**	□Yes □No		□Yes □No		Days

PLEASE FILL OUT PAIN DIAGRAM FOR YOUR NEW LOW BACK

23. What do you think caused this New Pain?

______Unsure
______ Accident outside of work (slip, trip, fall, MVA, etc.)
______ Something outside of work (NOT an accident)
______ Accident at work (slip, trip, fall, MVA, etc.)
______ Something at work (Not an accident)
______ Relapse or aggrivation of previously reported pain

24. When did the Pain occur?

(Don't answer if cause =”Unsure”
______Sudden (within 30 minutes)
______Same dau (after 30 mimutes)
______Next day or after
______Don't Know

25. Did a job evaluation team study the part of your job related to when/where you were injured?

_______Yes
_______No
_______Not Applicable

26. Do you have any new NUMBNESS/TINGLING in your:

☑	**Body Part**	**Number of days you have had N/T.**	**How often do you have N/T upon awakening in the morning?**	**How often do you have N/T at night?**
☐	R. Thigh (R)			
☐	R. Lateral Calf (Z)			
☐	R. Medial Calf (Y)			

☐	R. Foot (S)			
☐	R. Toes (U)			
☐	L. Thigh (Q)			
☐	L. Lateral Calf (W)			
☐	L. Medial Calf (X)			
☐	L. Foot (T)			
☐	L. Toes (V)			

27. What do you think caused this NUMBNESS/TINGLING?
    ______Unsure
    ______ Accident outside of work (slip, trip, fall, MVA, etc.)
    ______ Something outside of work (NOT an accident)
    ______ Accident at work (slip, trip, fall, MVA, etc.)
    ______ Something at work (Not an accident)
    ______ Relapse or aggrivation of previously reported pain

28. When did the Numbness/Tingling occur?
    (Don't answer if cause =”Unsure”
    ______Sudden (within 30 minutes)
    ______Same dau (after 30 mimutes)
    ______Next day or after
    ______Don't Know

29. Does the N/T occur at the same time as the low back pain?
    ______Yes
    ______No

30. Since your follow-up ____ days ago, have you been on light duty because of Low Back Pain?
    ______Yes
    ______No

    If yes, How many days ago or what day did you begin light duty?
    ________________________   ____________

month day year days ago

When did you end or will you end working light duty?

____
month day year

31. Since your last follow-up ____ days ago, have you had any lost duty days due to Low Back Pain?
______Yes
______No

If Yes, How many lost duty days did you have?
______days

32. Are you taking any medication or getting any treatment for your pain or numbness/tingling?
______Yes
______No

If YES, What types of treatment are you using?

☑	**Mid-Back**	Yes / No
☐	Back Surgery	
☐	Injection in the back	
☐	Back Brace	
☐	Chiropractor	
☐	Physician	
☐	PT or OT	
☑	**Low Back**	Yes / No
☐	Back Surgery	Yes / No
☐	Injection in the back	Yes / No
☐	Back Brace	Yes / No
☐	Chiropractor	Yes / No

☐	Physician	Yes / No
☐	PT or OT	Yes / No
	**Other**	
☐	NSAID or OTC Meds	Yes / No
☐	Prescription Meds not NSAID or Narcotic	Yes / No
☐	Prescription, Narcotic / painkillers	Yes / No
☐	Light Duty	Yes / No
☐	Aerobic Exercise	Yes / No
☐	Strength Exercise	Yes / No
☐	Stretching	Yes / No
☐	Other	Yes / No
☐	Other	Yes / No

33. Since we saw you _____ days ago, Have you:

Changed jobs / lines	Yes/No
Changed the number of hours you work	Yes / No
Started using new / different equipment	Yes / No
Changed your rotation schedule	Yes / No
Dhanged your production rate	Yes / No
Other	Yes / No

34. Does the participant complain of a "tired back" even though there is no new or recurrent Low Back Pain?
______Yes
______No

35. If you were absent at our last follow-up visit, what day did you return to work?

____
Month Day Year

36. If you were not here the last time we collected monthly follow-up data, why were you absent?
______ill
______Vacation
______Pregnancy
______Light Duty
______Injury
______Surgery or medical appt.
______Other

Monthly Follow-Up Low Back Pain Diagram #1

Typical Low Back Pain in the past month

This is to be completed by anyone indicating pain in Lumbar Immediately Paraspinal **(M),** Right Lumbar **(N)** Left Lumber **(L),** Right Gluteal **(P),** and/or Left Gluteal **(O)**

98. **Circle** the area of the low back where you typically experienced pain **in the past month**.

99. **Mark with an "X"** on the diagram where you experienced the **WORST** pain.

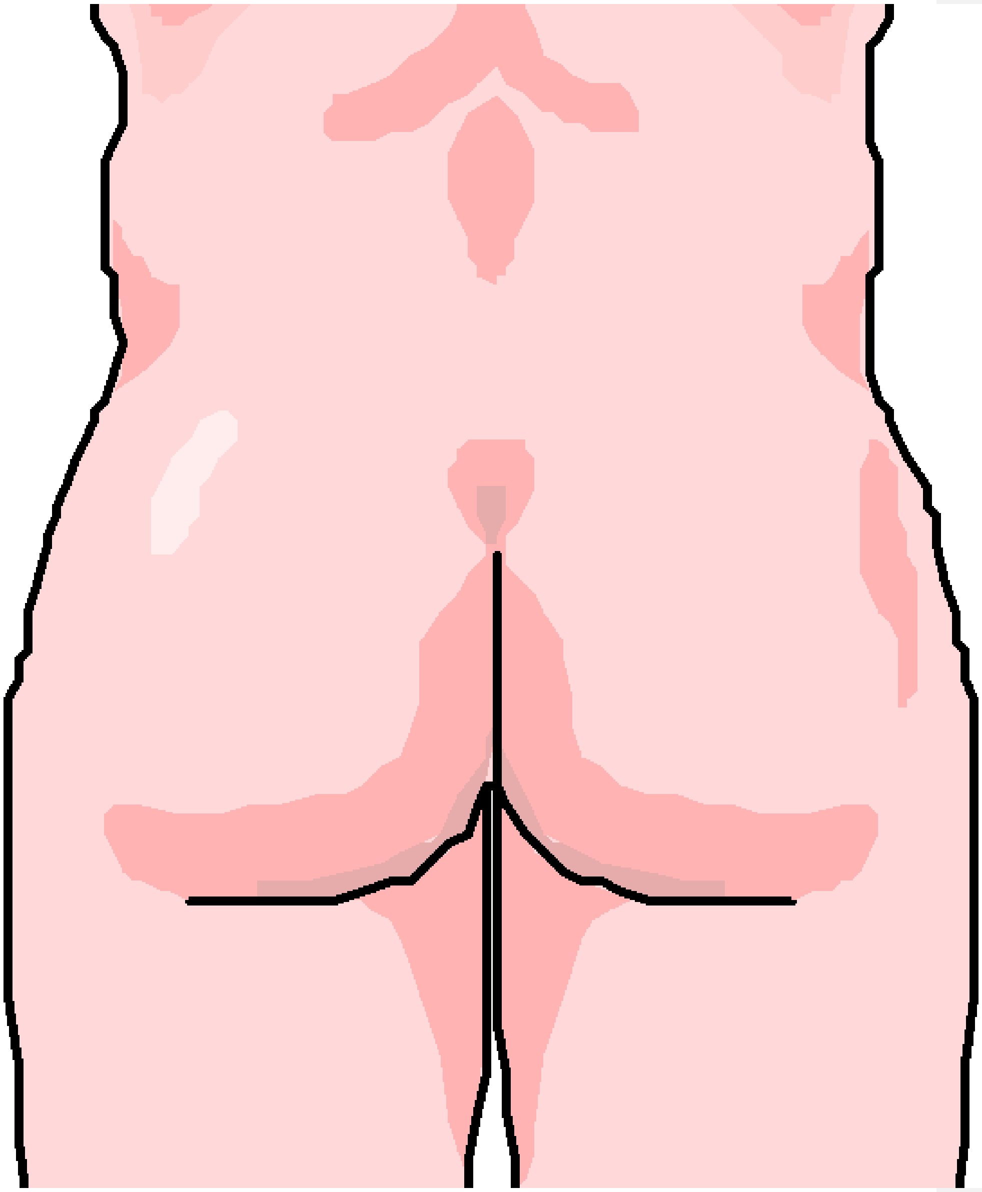

A.5. Exit Interview

An Ergonomic-Epidemiologic Study
of the Distal Upper Extremity and Low Back:
Exit Questionnaire

Directions:

Please answer each question by pointing the arrow with the mouse and clicking with you index finger to either mark "yes" or "no" or to fill in a blank. If you need help or have any questions please ask one of our research assistants. We're happy to help!

Code # ___________________________
Date: ____________________________

An Ergonomic-Epidemiologic Study of the Low Back: Baseline Questionnaire

1. Do you walk on a regular basis **at least twice a month** outside of work?
   ____Yes ____No

a. If yes,

Type of Exercise	Number of months per year	Average number of times per week	Average number of minutes each time
Walking	Months	Per week	Minutes

b. Do you do any other exercise outside of work (for example, bicycling, basketball, skiing, or swimming) at least twice a month? ____Yes ____No

a. Which of the following types of exercise (outside of work) do you do?

Type of Exercise	Yes	No	Number of months per year	Average number of times per week	Average number of minutes each time
Aerobics, Jazzercise			Months	Per week	Minutes
Running, Jogging			Months	Per week	Minutes
Bowling			Months	Per week	Minutes
Bicycling			Months	Per week	Minutes
Swimming			Months	Per week	Minutes
Weight Lifting			Months	Per week	Minutes
Baseball			Months	Per week	Minutes
Basketball			Months	Per week	Minutes
Football			Months	Per week	Minutes
Soccer			Months	Per week	Minutes
Racquetball			Months	Per week	Minutes
Handball			Months	Per week	Minutes
Tennis			Months	Per week	Minutes

Snow Skiing or Snowboarding			Months	Per week	Minutes
Water Skiing or Wave Runner			Months	Per week	Minutes
Hunting			Months	Per week	Minutes
Fishing			Months	Per week	Minutes
Martial Arts (Karate, Judo, etc.)			Months	Per week	Minutes
Other (please list)			Months	Per week	Minutes
Other (please list)			Months	Per week	Minutes

100. Has anyone in your family (biological parents and/or siblings) ever had a pinched nerve, Sciatica, or **Lumbar Radiculopathy** in the lower back? _____ Yes _____ No

101. Has any one in your family (biological parents and/or siblings) ever been diagnosed with **Carpal Tunnel Syndrome**? _____ Yes _____ No

102. How often do you have **family problems** that irritate or bother you?
____ Never
____ Sometimes
____ Often
____ Always

103. How often during the past month have you **felt uneasy?**
____ Never
____ Sometimes
____ Often
____ Always

104. How often during the past month have **you felt tense?**
____ Never
____ Sometimes
____ Often
____ Always

105. How often during the past month have you **felt "down", blue or depressed?**
____ Never
____ Sometimes
____ Often
____ Always

106. How often during the past month have you felt **nervous or anxious?**
____ Never
____ Sometimes
____ Often
____ Always

107. All in all, how satisfied are you with your job?
____ Very satisfied
____ Somewhat satisfied
____ A little satisfied
____ Not at all satisfied

108. I have an ache and/or pain, in any body part approximately about how often? ____% of days
____ Never (0%)
____ Rarely (0-20%)
____ Sometimes (20-40%)
____ About Half the Time (40-60%)
____ Often (60-80%)
____ Very often (80-100%)
____ Always (100%)

109. How often do you take Tylenol or acetaminophen?
a. ____ Never or almost never (0-6 times a year)
b. ____ Rarely (less than once a month, 7-11 times a year)
c. ____ 1-4 times a month (12-50 times a year)
d. ____ 1-3 times a week (50-150 times a year)
e. ____ 4-6 times a week (150-300 times a year)
f. ____ Daily (7 times a week, 300-365 times a year)
g. ____ More than once a day (more than 365 times a year)
h. ____ I don't know (a research assistant will help)

i. Is this a prescription or over-the-counter?
   i. ____ Prescription
   ii. ____ Over the counter

110. How often do you take ibuprofen, Motrin, Advil, Naprosyn, or aspirin?
a. ____ Never or almost never (0-6 times a year)
b. ____ Rarely (less than once a month, 7-11 times a year)
c. ____ 1-4 times a month (12-50 times a year)
d. ____ 1-3 times a week (50-150 times a year)
e. ____ 4-6 times a week (150-300 times a year)
f. ____ Daily (7 times a week, 300-365 times a year)
g. ____ More than once a day (more than 365 times a year)
h. ____ I don't know (a research assistant will help)

i. Is this a prescription or over-the-counter?
   i. ____ Prescription
   ii. ____ Over the counter

111. How often do you take opioids such as Lortab, hydrocodone, Tramadol, Demerol or codeine?

a. ____ Never or almost never (0-6 times a year)
b. ____ Rarely (less than once a month, 7-11 times a year)
c. ____ 1-4 times a month (12-50 times a year)
d. ____ 1-3 times a week (50-150 times a year)
e. ____ 4-6 times a week (150-300 times a year)
f. ____ Daily (7 times a week, 300-365 times a year)
g. ____ More than once a day (more than 365 times a year)
h. ____ I don't know (a research assistant will help)

i. Is this a prescription or over-the-counter?
    i. ____ Prescription
    ii. ____ Over the counter

112. In the past **year**, have you had pain below your knee that you think is from a problem in your back?
a. Yes, I have had pain below my knee that I think is from a problem in my back.
    i. I would rate it a pain of ___/10 at its worst
b. No, I have not had any pain below my knee or foot in the past year that I think is from a problem in my back.

113. In the past month, have you had pain in your knee that is not related to an accident?
a. Yes,
    i. I would rate it a pain of ___/10 at its worst
b. No, I have not had any pain in my knee in the past month.

114. In the past month, have you had pain in your ankle or foot that is not related to an accident?

a. Yes, in my ankle
    i. I would rate it a pain of ___/10 at its worst
b. Yes, in my foot
    i. I would rate it a pain of ___/10 at its worst
c. No, I have not had any pain in my ankle or foot in the past month.

115. How many times do you eat **restaurant or fast food** in a typical week? (For example Chili's™, McDonald's™, Burger King™, or your local diner. Take home leftovers count twice)
a. 0; 1; 2; 3; 4; 5; 6; 7; more than 7

116. When eating **restaurant food**, do you eat all the food served to you at one time?
a. Never
b. Rarely
c. Occasionally
d. Sometimes
e. Often
f. Usually
g. Always

117. How many times do you typically eat breakfast in **one week** (7 days)?
   a. 0; 1; 2; 3; 4; 5; 6; 7; more than 7

118. How many times do you typically eat a serving of vegetables in **one day** (For example salad, V-8 Juice, carrots, broccoli, etc.)?
   a. 0; 1; 2; 3; 4; 5; 6; 7; more than 7

119. How many times do you typically eat a serving of fruit in **one day** (For example, an apple, an orange, raisins, fruit juice, a hand full of grapes, etc)?
   a. 0; 1; 2; 3; 4; 5; 6; 7; more than 7

120. How do you feel about how much fruit you eat in one day?
   a. I do not plan to start eating more or less fruit.
   b. I plan to start eating more fruit in the next 6 months.
   c. I plan to start eating more fruit in the next month.
   d. I have been trying to eat more fruit recently (within 6 months).
   e. I have been trying to eat more fruit for more than 6 months.
   f. I plan to start eating less fruit.

121. Overall, when you think about the foods you ate over the past 12 months, would you say your diet was high, medium, or low in fat (e.g. butter, cook with oil, etc.)?
   a. High
   b. Medium
   c. Low

122. How do you feel about how much fat you eat?
   a. I do not plan on trying to eat more or less fat
   b. I plan to try to eat less fat in the next 6 months.
   c. I plan to start eating less fat in the next month.
   d. I have been trying to eat less fat recently (within 6 months).
   e. I have been trying to eat less fat for over 6 months.
   f. I plan to start eating more fat.
   g. I don't know what the fat content of my diet is.

123. Do you know what your current **TOTAL Blood Cholesterol** is?
   a. Yes, it is about ______ mg/dl
   b. No

124. Do you think your **Total Cholesterol** is:
   a. Good
   b. Not good
   c. I am unsure or don't know.

125. Do you currently take Cholesterol –Lowering Medication?
   a. Yes____

i. If Yes: About how long have you been taking that medication? ______ Years

b. No____

126. Do you know what your Body Mass Index (BMI) is?
  a. Yes, it is about _______ kg/m^2
  b. No

127. Do you believe you are:
  a. Underweight
  b. Normal Weight
  c. Overweight
  d. Obese
  e. I am not sure or don't know

128. Throughout the duration of this study, have you received any education (your doctor, the internet, magazines etc) in:
  a. Weight management Yes____ No____
  b. Diet/ Nutrition Yes____ No____
  c. Physical Fitness Yes____ No____

129. Regular physical exercise is defined as being active on 3 or more days a week. **Do you participate in <u>regular physical exercise</u>?**
  a. I don't and I am not thinking about starting in the near future.
  b. I don't but I plan to start in the near future.
  c. I don't but I do at least some physical exercise once or twice per week.
  d. I do and have done so for less than 6 months.
  e. I do and have done so for longer than 6 months.

130. Which statement best describes how you feel about your **<u>weight</u>**:
  a. My weight is about right and I am not planning on trying to change it.
  b. I want to lose weight, but I don't know where to start.
  c. I have decided to lose weight and I have a plan to start in the next 6 months.
  d. I plan on starting to make changes (diet, exercise etc) within the next month.
  e. I have recently made changes to lose weight (within 6 months).
  f. I have been making changes to lose weight for longer than 6 months.
  g. I am trying to gain weight.

Please place a checkmark in the box corresponding with how you feel about each of the following statements.

		Almost always	Some of the time	Hardly ever
131.	I am satisfied that I can turn to a fellow worker for help if something is troubling me.			

132.	I am satisfied that my fellow workers accept and support my new ideas and thoughts.			
133.	I am satisfied with how well I get along with my closest or immediate supervisor.			

Modified Zung Depression Index

Please indicate for each of the following questions which answer best describes how you have been feeling recently.	Rarely or none of the time (less than 1 day per week)	Some or little of the time (1–2 days per week)	A moderate amount of time (3–4 times per week)	Most of the time (5–7 days per week)
134. I feel that nobody cares				

135. Altogether, about how much time do you spend **sitting while at work**?

☐ ½ hour ☐ 1 hour ☐ 1½ hours ☐ 2 hours ☐ 2½ hours ☐ 3 hours

☐ 3½ hours ☐ 4 hours ☐ 4½ hours ☐ 5 hours ☐ 5½ hours ☐ 6 hours

☐ 6½ hours ☐ 7 hours ☐ 7½ hours ☐ 8 hours ☐ 8½ hours ☐ 9 hours

☐ 9½ hours ☐ 10 hours ☐ 10½ hours ☐ 11 hours ☐ 11½ hours ☐ 12 hours

136. Do you have high foot arches? Yes / No / Unsure

137. Do you have flat feet? Yes / No / Unsure

The following statements ask you to think about physical pain you have or have had and how it relates to God and religion. Please consider your answers to these questions regardless of whether or not you have received any type of medical treatment.

138. On average, how often do you attend religious services?
   a) More than once a week
   b) Once a week

c) 2-3 times a month
d) Once a month
e) Every other month
f) 1-3 times a year
g) Never
h) Don't know
i) Decline to answer

139. On average, how often do you communicate with God? (Prayer at bedtime, meals, family time, etc):

a) More than once a day
b) Daily
c) 2-3 times a week
d) 2-3 times a month
e) Less than once a month
f) Never
g) Don't know
h) Decline to answer

140. Generally, when thinking about your physical pain, which of the following statements describes how you ***most often*** think about physical pain in relation to God;

a) Wondered if it was a punishment from God
b) Thought that God might be trying to strengthen me
c) Neither

141. Generally, when thinking about your physical pain, which of the following statements describes how you ***most often*** try to alleviate physical pain in relation to God;

a. Only pray for relief
b. Make a bargain with God to relieve my pain
c. Make a plan with God to relieve my pain
d. None of the above

142. Generally, when thinking about your physical pain, which of the following statements describes how you ***most often*** try to alleviate physical pain in regards to religious members and/or leaders;

a. Look to religious members and/or leaders for spiritual support (e.g. prayers of healing)
b. Look to religious members and/or leaders for social support (e.g. bring you food or medicine, give advice on treatment or doctors, etc.)
c. Neither

143. I try hard to live all my life according to religious beliefs.

0 (I never live my life according to religious beliefs)
1
2

3
4
5
6
7
8
9
10 (I always live my life according to religious beliefs)

144. Other things in life are more important than religion and/or God.

0 (Nothing else is more important than religion and/or God)
1
2
3
4
5
6
7
8
9
10 (Everything is more important than religion and/or God)

145. What is your religious affiliation?
____ Agnostic
____ Atheist
____ Buddhist
____ Catholic
____ Church of Jesus Christ of Latter Day Saints
____ Hindu
____ Islamic
____ Jewish
____ Non-denominational (e.g. Church of Christ, Assembly of God, etc.)
____ Orthodox
____ Protestant (e.g. Presbyterian, Methodist, Baptist, etc.)
____ Unaffiliated
____ Other, (please specify) ____________________________________
____ Decline to answer

Thank you for completing the questionnaire.
