# Supplementary material for: Study protocol title: a prospective cohort study of low back pain
Source: BMC Musculoskelet Disord. 2013 Mar 7;14:84. doi: 10.1186/1471-2474-14-84 (PMC3599364; doi:10.1186/1471-2474-14-84)
Supplement: Additional file 2: Appendix B — Job Specific Data. [file 1471-2474-14-84-S2.pdf]

APPENDIX B

B.1. Position/Worker Specific Data Form ....
B.2. Job Summary Data Form..
B.3. Job Specific Data Form ....
B.4. Lifting/Lowering Steady Data Form ....
B.5. Lifting/Lowering Variable Data Form......................................
B.6. Pushing/Pulling Data Form...
B.7. Walking/Carrying Data Form ....
B.8. Lifting/Lowering Tasks Data Form...
B.9. Pushing/Pulling Tasks Data Form...
B.10. Walking/Carrying Tasks Data Form ....

B.1. Position/Worker Specific Data Form

Position / Worker Specific Data Form (Form A)

1. Subject I.D. ____________________________ <AA0001>
2. Subject Name: __________________________
3. ☐Male ☐Female
4. Age: __________ <years>
5. Facility: ______________________________
6. Date: ______________________________ <MM / DD / YYYY>
7. Time (24hr): ________________________ <2:30 pm = 14:30, midnight = 00:00>
8. Analyst #1: ___________________________
9. Analyst #2: ___________________________

Position Information

10. Line / Department Title: ____
11. Position Title: ____
12. Position Description: ____

____

____

____

13. Typical Shift ***Start*** Time (24hr): ___________
14. Typical Shift ***End*** Time (24hr): ___________
15. Break-Time (Minutes/day): ________ Minutes <Total of Lunch & Breaks / Day>
16. # of ***Distinct*** Jobs Rotated to: ___________
17. Average Work ***Hours***/Week: ____________
18. Maximum Work ***Hours***/Week: ___________
19. Average Work ***Days***/Week: _____________
20. Maximum Work ***Days***/Week: ____________
21. Note Unusual Schedule Here: ____

____

22. Jobs Included in Position:

Job #	Job Title/Description	PACE Self, Line, Piece Rate	LBS/Rotation (lbs/t)	Continuous Duration (Hours, t)	Total Hours/ Day (Σt, Hours)	Typical Work % of Week	# of Times/ Month (Peak Exposures Only)
1		S L P					
2		S L P					
3		S L P					
4		S L P					
5		S L P					
6		S L P					
7		S L P					
8		S L P					

B.2. Job Summary Data Form

Subject ID: ______________________________

Worker Information

23. Prior Work Experience; Maximum of 10 Years ***OR*** Maximum of 5 Jobs[1]:
(Include Current Position and *significantly* different prior positions with *present* employer first)

Position	Title / Description	Years	Average Low Back Borg Rating
Current			
Prior #1			
Prior #2			
Prior #3			
Prior #4			
Prior #5			

24. Do you currently work on a second job outside of this facility? ☐Yes ☐No

25. If Yes, 2nd Job Title/Description: ____

____

26. Average Hours/Week on 2nd Job: _________ 27. Number of Years on 2nd Job: ____________

28. Low Back Overall (Average) Borg Rating for 2nd Job: ___________________________________

	Worker Estimated Rating (Stress) (Low Back Only)	Worker Rating @ Beg/End (Stress) (Low Back Only)
29. Overall Worker Rating at ***Beginning*** of Shift[3] (Borg CR-10)		
30. Overall Worker Rating at ***End*** of Shift[3] (Borg CR-10)		

31. Analyst Notes (Optional): ____

____

____

____

Job Summary Data Form (Form B)

1. Subject I.D. ______________________________ 2. Job # (From "Position Data Form, Field #22"): ________
<From Position Form, Field #1>

3. Facility: ______________________________ 4. Date: ______________________________
<MM / DD / YYYY>

5. Data Form Inventory

Data Form 'Name'	Form 'Letter'	Not Used	# of TOTAL Pages
Job Specific Data Form	C		1
Steady State Lifting/Lowering	D		
Variable State Lifting/Lowering	E		
Pushing/Pulling	F		
Walking/Carrying/Holding	G		

6. Summary Notes/Data (include "to-do" for analysis and "issues" with data):

7. Jobs Considered Identical for Data Analysis Purposes

Name	Location	Collected?	Notes:

8. Time Adjustments for Motion Counts / Energy Expenditure

Tape Code	Video Code	Segment Length	Actual Length	Ratio

9. Motion Counts: (Load = >4lbs/Hand)	*W/ Load*	*W/O Load*
# Trunk Flexions (≥30°)		
# Trunk Rotations (≥±45°)		
# Lateral Bend (≥10°)		
# 180° Turns		

Form # LBS05042004

B.3. Job Specific Data Form

Job Specific Data Form (Form C)

1. Subject I.D. ______________________________
   <From Position Form, Field #1>
2. Analyst Name(s): ______________________
3. Observation Time (minutes): ______________
4. Job ID (Plant Official): ______________________
5. Facility: ______________________________
6. Time (24hr): ____________________________
   <2:30 pm = 14:30, midnight = 00:00>
7. Date: ________________________________
   <MM / DD / YYYY>
8. Job # (From "Position Data Form, Field #22"): ________

Job Overview

9. Specify the ***Percentage of Time*** Spent in EACH of the Sub-Activities Listed Below:

Manual Material Handling	Assembly (Specify Light or Heavy Assembly)	Paperwork (with or without Computer)	Fork-Truck Riding	Resting/Waiting
________%	________%	________%	________%	________%
☐Sitting ☐Standing	☐Sitting ☐Standing<br>☐Light ☐Heavy	☐Sitting ☐Standing	☐Sitting ☐Standing	☐Sitting ☐Standing

10. Specify the ***Low-Back Borg CR-10 Rating*** for EACH of the Sub-Activities Listed Below:

Manual Material Handling		Assembly		Paperwork (with or without Computer)		Fork-Truck Riding		Resting/Waiting	
Worker	Analyst	Worker	Analyst	Worker	Analyst	Worker	Analyst	Worker	Analyst
CR-10	CR-10	CR-10	CR-10	CR-10	CR-10	CR-10	CR-10	CR-10	CR-10

11. Specify the Single, ***Most Stressful Low-Back Task*** and Associated Borg CR-10 Rating Below:

Task	Worker Rating	Analyst Rating
	CR-10	CR-10

12. Worker Postures (Standing + Sitting = 100%; Back Bent and Squatting are expressed as a sub percentage of standing or sitting (i.e. standing = 50%, Back Bent under standing = 100%, means that 100 percent of the time spent standing is in a bent posture.) NOTE: Walking is Counted as Standing

Standing %		Sitting %
Back Bent (>20°)	Squatting	Back Bent (>20°)
%	%	%

13. Worker Exposed to ***Whole Body Vibration***: ☐Yes ☐No
14. Worker Use of ***Anti-Fatigue*** Mats: ☐Yes ☐No
    Anti-Fatigue Mat ***Usage Time***: _____%
15. Use of "***Insoles***" in shoes: ☐Yes ☐No
16. Worker Uses ***Back Belt***: ☐Yes ☐No
    Back Belt ***Available***: ☐Yes ☐No
    Has Back Belt ***Training***: ☐Yes ☐No
17. Floor: ☐Normal ☐Uneven ☐Slippery
    NOTE: Floors can be Uneven & Slippery
18. Work Space (MMH): ☐Open ☐Obstructed
    Type of Obstruction: __________________
19. Typical Plant ***Temperature***:

January	July
°C	°C

20. Typical Plant ***Humidity***:

January	July
%R.H.	%R.H.

Job #____-1

Form #: LBJ05072004

B.4. Lifting/Lowering Steady Data Form

Lifting/Lowering Steady (Form D) **Subject # ____________ Job # _____**

Normal Carry Height ____________ in

Object Description:____________________________ Object Location:____________________________

☐ Lift<br>☐ Lower	W (lbs)	Box Width (in)	H_{OL} (in)	H_{OR} (in)	V_O (in)	H_{DL} (in)	H_{DR} (in)	V_D (in)

Object Description:____________________________ Object Location:____________________________

☐ Lift<br>☐ Lower	W (lbs)	Box Width (in)	H_{OL} (in)	H_{OR} (in)	V_O (in)	H_{DL} (in)	H_{DR} (in)	V_D (in)

Object Description:____________________________ Object Location:____________________________

☐ Lift<br>☐ Lower	W (lbs)	Box Width (in)	H_{OL} (in)	H_{OR} (in)	V_O (in)	H_{DL} (in)	H_{DR} (in)	V_D (in)

Object Description:____________________________ Object Location:____________________________

☐ Lift<br>☐ Lower	W (lbs)	Box Width (in)	H_{OL} (in)	H_{OR} (in)	V_O (in)	H_{DL} (in)	H_{DR} (in)	V_D (in)

Object Description:____________________________ Object Location:____________________________

☐ Lift<br>☐ Lower	W (lbs)	Box Width (in)	H_{OL} (in)	H_{OR} (in)	V_O (in)	H_{DL} (in)	H_{DR} (in)	V_D (in)

Object Description:____________________________ Object Location:____________________________

☐ Lift<br>☐ Lower	W (lbs)	Box Width (in)	H_{OL} (in)	H_{OR} (in)	V_O (in)	H_{DL} (in)	H_{DR} (in)	V_D (in)

Object Description:____________________________ Object Location:____________________________

☐ Lift<br>☐ Lower	W (lbs)	Box Width (in)	H_{OL} (in)	H_{OR} (in)	V_O (in)	H_{DL} (in)	H_{DR} (in)	V_D (in)

Object Description:____________________________ Object Location:____________________________

☐ Lift<br>☐ Lower	W (lbs)	Box Width (in)	H_{OL} (in)	H_{OR} (in)	V_O (in)	H_{DL} (in)	H_{DR} (in)	V_D (in)

Object Description:____________________________ Object Location:____________________________

☐ Lift<br>☐ Lower	W (lbs)	Box Width (in)	H_{OL} (in)	H_{OR} (in)	V_O (in)	H_{DL} (in)	H_{DR} (in)	V_D (in)

Object Description:____________________________ Object Location:____________________________

☐ Lift<br>☐ Lower	W (lbs)	Box Width (in)	H_{OL} (in)	H_{OR} (in)	V_O (in)	H_{DL} (in)	H_{DR} (in)	V_D (in)

Date: ________________ **Page # ____ of ____**

Form #LBLLS05042004

B.5 Lifting/Lowering Variable Data Form

Lifting/Lowering Variable (Form E)

Subject # ____________ Job # _____

Normal Carry Height ____________ in

1.

W (lbs)	Width (in)	H_o (in)	V_o (in)	H_D (in)	V_D (in)

2.

W (lbs)	Width (in)	H_o (in)	V_o (in)	H_D (in)	V_D (in)

3.

W (lbs)	Width (in)	H_o (in)	V_o (in)	H_D (in)	V_D (in)

4.

W (lbs)	Width (in)	H_o (in)	V_o (in)	H_D (in)	V_D (in)

5.

W (lbs)	Width (in)	H_o (in)	V_o (in)	H_D (in)	V_D (in)

6.

W (lbs)	Width (in)	H_o (in)	V_o (in)	H_D (in)	V_D (in)

7.

W (lbs)	Width (in)	H_o (in)	V_o (in)	H_D (in)	V_D (in)

8.

W (lbs)	Width (in)	H_o (in)	V_o (in)	H_D (in)	V_D (in)

9.

W (lbs)	Width (in)	H_o (in)	V_o (in)	H_D (in)	V_D (in)

10.

W (lbs)	Width (in)	H_o (in)	V_o (in)	H_D (in)	V_D (in)

11.

W (lbs)	Width (in)	H_o (in)	V_o (in)	H_D (in)	V_D (in)

12.

W (lbs)	Width (in)	H_o (in)	V_o (in)	H_D (in)	V_D (in)

13.

W (lbs)	Width (in)	H_o (in)	V_o (in)	H_D (in)	V_D (in)

14.

W (lbs)	Width (in)	H_o (in)	V_o (in)	H_D (in)	V_D (in)

15.

W (lbs)	Width (in)	H_o (in)	V_o (in)	H_D (in)	V_D (in)

16.

W (lbs)	Width (in)	H_o (in)	V_o (in)	H_D (in)	V_D (in)

17.

W (lbs)	Width (in)	H_o (in)	V_o (in)	H_D (in)	V_D (in)

18.

W (lbs)	Width (in)	H_o (in)	V_o (in)	H_D (in)	V_D (in)

19.

W (lbs)	Width (in)	H_o (in)	V_o (in)	H_D (in)	V_D (in)

20.

W (lbs)	Width (in)	H_o (in)	V_o (in)	H_D (in)	V_D (in)

21.

W (lbs)	Width (in)	H_o (in)	V_o (in)	H_D (in)	V_D (in)

22.

W (lbs)	Width (in)	H_o (in)	V_o (in)	H_D (in)	V_D (in)

23.

W (lbs)	Width (in)	H_o (in)	V_o (in)	H_D (in)	V_D (in)

24.

W (lbs)	Width (in)	H_o (in)	V_o (in)	H_D (in)	V_D (in)

Date: ________________ **Page # ____ of ____**

LBLLV05042004

B.6. Pushing/Pulling Data Form

Pushing/Pulling Notes (Form F)

Subject # ____________ Job # _____

Description:____

☐ Push	Initial Force (lbs)	Sustained Force (lbs)	Hand Height (in)	Distance (ft)	Time (sec)
☐ Pull					

Description:____

☐ Push	Initial Force (lbs)	Sustained Force (lbs)	Hand Height (in)	Distance (ft)	Time (sec)
☐ Pull					

Description:____

☐ Push	Initial Force (lbs)	Sustained Force (lbs)	Hand Height (in)	Distance (ft)	Time (sec)
☐ Pull					

Description:____

☐ Push	Initial Force (lbs)	Sustained Force (lbs)	Hand Height (in)	Distance (ft)	Time (sec)
☐ Pull					

Description:____

☐ Push	Initial Force (lbs)	Sustained Force (lbs)	Hand Height (in)	Distance (ft)	Time (sec)
☐ Pull					

Description:____

☐ Push	Initial Force (lbs)	Sustained Force (lbs)	Hand Height (in)	Distance (ft)	Time (sec)
☐ Pull					

Description:____

☐ Push	Initial Force (lbs)	Sustained Force (lbs)	Hand Height (in)	Distance (ft)	Time (sec)
☐ Pull					

Description:____

☐ Push	Initial Force (lbs)	Sustained Force (lbs)	Hand Height (in)	Distance (ft)	Time (sec)
☐ Pull					

Description:____

☐ Push	Initial Force (lbs)	Sustained Force (lbs)	Hand Height (in)	Distance (ft)	Time (sec)
☐ Pull					

Description:____

☐ Push	Initial Force (lbs)	Sustained Force (lbs)	Hand Height (in)	Distance (ft)	Time (sec)
☐ Pull					

Description:____

☐ Push	Initial Force (lbs)	Sustained Force (lbs)	Hand Height (in)	Distance (ft)	Time (sec)
☐ Pull					

Date: ________________ Page # ____ of ____

LBDPP04052004

B.7. Walking/Carrying Data Form

Walking/Carrying Notes (Form G) **Subject # ____________ Job # _____**

Description:____

☐ Walk	Weight (lbs)	Distance (ft)	Time (sec)
☐ Carry			

Description:____

☐ Walk	Weight (lbs)	Distance (ft)	Time (sec)
☐ Carry			

Description:____

☐ Walk	Weight (lbs)	Distance (ft)	Time (sec)
☐ Carry			

Description:____

☐ Walk	Weight (lbs)	Distance (ft)	Time (sec)
☐ Carry			

Description:____

☐ Walk	Weight (lbs)	Distance (ft)	Time (sec)
☐ Carry			

Description:____

☐ Walk	Weight (lbs)	Distance (ft)	Time (sec)
☐ Carry			

Description:____

☐ Walk	Weight (lbs)	Distance (ft)	Time (sec)
☐ Carry			

Description:____

☐ Walk	Weight (lbs)	Distance (ft)	Time (sec)
☐ Carry			

Description:____

☐ Walk	Weight (lbs)	Distance (ft)	Time (sec)
☐ Carry			

Description:____

☐ Walk	Weight (lbs)	Distance (ft)	Time (sec)
☐ Carry			

Date: ________________ **Page # ____ of ____** LBDWC05042004

B.8. Lifting/Lowering Tasks Data Form

Lifting/Lowering Tasks (Combined Form) **Subject # __________ Job # ____**

1. *Task #*____ 2. *Description:*____

3. ☐Lift ☐Lower 4. ☐Box ☐Bag ☐Other____

5. Separate Hands: ☐Yes ☐No 6. ☐Even ☐Uneven | ☐Stable ☐Unstable

7. *Hands:* ☐Left ☐Right ☐Both 8. *Grasp:* ☐Good ☐Fair ☐Poor

9. *Technique:* ☐Stoop ☐Semi ☐Squat ☐Side ☐Stand

10. *Object Dimensions:* W____in, L____in, H____in

11. *Observation Duration:*____min 12. *Number of Lift/Lowers:*____

13. *Total Weight/Lift:*____lbs *SCD?* ☐Yes ☐No

V_{OL}	in	V_{OR}	in	V_{DL}	in	V_{DR}	in
H_{OL}	in	H_{OR}	in	H_{DL}	in	H_{DR}	in
L_{OL}	in	L_{OR}	in	L_{DL}	in	L_{DR}	in
D	in	A_O	°	A_D	°		

1. ***Task #***____ 2. ***LIFT/LOWER Biomechanical Analysis***

3. ***Object Weight:*** ____lbs 4. ***Hands:* L R B**

5. ***Limb Angles:***

	Left		Right	
	Horz.	*Vert.*	*Horz.*	*Vert.*
Upper Arm	°	°	°	°
Lower Arm	°	°	°	°
Upper Leg	°	°	°	°
Lower Leg	°	°	°	°

6. ***Trunk Angles:***

Flexion	°
Axial Rotation R L	°
Lateral Bending R L	°

NOTE: Lifting and then lowering are 2 seperate tasks (one lifting and one lowering).

1. *Task #*____ 2. *Description:*____

3. ☐Lift ☐Lower 4. ☐Box ☐Bag ☐Other____

5. Separate Hands: ☐Yes ☐No 6. ☐Even ☐Uneven | ☐Stable ☐Unstable

7. *Hands:* ☐Left ☐Right ☐Both 8. *Grasp:* ☐Good ☐Fair ☐Poor

9. *Technique:* ☐Stoop ☐Semi ☐Squat ☐Side ☐Stand

10. *Object Dimensions:* W____in, L____in, H____in

11. *Observation Duration:*____min 12. *Number of Lift/Lowers:*____

13. *Total Weight/Lift:*____lbs *SCD?* ☐Yes ☐No

V_{OL}	in	V_{OR}	in	V_{DL}	in	V_{DR}	in
H_{OL}	in	H_{OR}	in	H_{DL}	in	H_{DR}	in
L_{OL}	in	L_{OR}	in	L_{DL}	in	L_{DR}	in
D	in	A_O	°	A_D	°		

1. ***Task #***____ 2. ***LIFT/LOWER Biomechanical Analysis***

3. ***Object Weight:*** ____lbs 4. ***Hands:* L R B**

5. ***Limb Angles:***

	Left		Right	
	Horz.	*Vert.*	*Horz.*	*Vert.*
Upper Arm	°	°	°	°
Lower Arm	°	°	°	°
Upper Leg	°	°	°	°
Lower Leg	°	°	°	°

6. ***Trunk Angles:***

Flexion	°
Axial Rotation R L	°
Lateral Bending R L	°

NOTE: Lifting and then lowering are 2 seperate tasks (one lifting and one lowering).

Date: ______________ Page # ____ of ____

Form #: LBLC03222004

B.9. Pushing/Pulling Tasks Data Form

Push/Pull Tasks (Combined Form) **Subject # ___________ Job # _____**

1. *Task #*_____ 2. *PUSH/PULL Biomechanical Analysis*

3. *Object Weight:* _____ lbs 4. *Hands:* *L R B*

5. *Limb Angles:*

	Left		Right	
	Horz.	Vert.	Horz.	Vert.
Upper Arm	°	°	°	°
Lower Arm	°	°	°	°
Upper Leg	°	°	°	°
Lower Leg	°	°	°	°

6. *Trunk Angles:*

Flexion	°
Axial Rotation R L	°
Lateral Bending R L	°

1. *Task #*_____ 2. *PUSH/PULL Biomechanical Analysis*

3. *Object Weight:* _____ lbs 4. *Hands:* *L R B*

5. *Limb Angles:*

	Left		Right	
	Horz.	Vert.	Horz.	Vert.
Upper Arm	°	°	°	°
Lower Arm	°	°	°	°
Upper Leg	°	°	°	°
Lower Leg	°	°	°	°

6. *Trunk Angles:*

Flexion	°
Axial Rotation R L	°
Lateral Bending R L	°

1. ***Task #***_____ 2. ***Description***: ______________________________

3. ***Activity***: ☐Push ☐Pull

4. ***Type of Cart***: ☐4W Cart ☐2W Hand Truck ☐Pallet Jack
☐Box ☐Other: ________________

5. ***Hands***: ☐Left ☐Right ☐Both 6. ***Height of Hands***: ________in

7. ***Posture***: ☐Lean Forward ☐Lean Back ☐Lean Side ☐Stand

8. ***Distance per Push/Pull***: _____ft 9. ***Time per Push/Pull***: ______sec

10. ***Initial Force***: ________lbs 11. ***Sustained Force***: ________lbs

12. ***Observation Duration***: ______min 13. ***Number of Push/Pulls***: _____

1. ***Task #***_____ 2. ***Description***: ______________________________

3. ***Activity***: ☐Push ☐Pull

4. ***Type of Cart***: ☐4W Cart ☐2W Hand Truck ☐Pallet Jack
☐Box ☐Other: ________________

5. ***Hands***: ☐Left ☐Right ☐Both 6. ***Height of Hands***: ________in

7. ***Posture***: ☐Lean Forward ☐Lean Back ☐Lean Side ☐Stand

8. ***Distance per Push/Pull***: _____ft 9. ***Time per Push/Pull***: ______sec

10. ***Initial Force***: ________lbs 11. ***Sustained Force***: ________lbs

12. ***Observation Duration***: ______min 13. ***Number of Push/Pulls***: _____

Date: ________________ **Page #** ____ **of** ____

Form #: LBUC03222004

B.10. Walking/Carrying Tasks Data Form

Walking/Carrying Tasks Subject # ____________ Job # ____

1. ***Task #*** ____ 2. ***Description:*** ____________
3. ***Activity:*** ☐Walk ☐Carry 14 ***Hands:*** ☐Left ☐Right ☐Both
5. ***Technique:*** ☐Waist ☐Thighs ☐Side ☐Other: ____________
6. ***Distance per Walk/Carry:*** ____ ft
7. ***Time per Walk/Carry:*** ____ sec
8. ***Object:*** ☐Box ☐Bag ☐Other: ____________
9. ***Object Weight:*** ____ lbs
10. ***Observation Duration:*** ____ min
11. ***Number of Walk/Carries:*** ____

1. ***Task #*** ____ 2. ***Description:*** ____________
3. ***Activity:*** ☐Walk ☐Carry 14 ***Hands:*** ☐Left ☐Right ☐Both
5. ***Technique:*** ☐Waist ☐Thighs ☐Side ☐Other: ____________
6. ***Distance per Walk/Carry:*** ____ ft
7. ***Time per Walk/Carry:*** ____ sec
8. ***Object:*** ☐Box ☐Bag ☐Other: ____________
9. ***Object Weight:*** ____ lbs
10. ***Observation Duration:*** ____ min
11. ***Number of Walk/Carries:*** ____

1. ***Task #*** ____ 2. ***Description:*** ____________
3. ***Activity:*** ☐Walk ☐Carry 14 ***Hands:*** ☐Left ☐Right ☐Both
5. ***Technique:*** ☐Waist ☐Thighs ☐Side ☐Other: ____________
6. ***Distance per Walk/Carry:*** ____ ft
7. ***Time per Walk/Carry:*** ____ sec
8. ***Object:*** ☐Box ☐Bag ☐Other: ____________
9. ***Object Weight:*** ____ lbs
10. ***Observation Duration:*** ____ min
11. ***Number of Walk/Carries:*** ____

1. ***Task #*** ____ 2. ***Description:*** ____________
3. ***Activity:*** ☐Walk ☐Carry 14 ***Hands:*** ☐Left ☐Right ☐Both
5. ***Technique:*** ☐Waist ☐Thighs ☐Side ☐Other: ____________
6. ***Distance per Walk/Carry:*** ____ ft
7. ***Time per Walk/Carry:*** ____ sec
8. ***Object:*** ☐Box ☐Bag ☐Other: ____________
9. ***Object Weight:*** ____ lbs
10. ***Observation Duration:*** ____ min
11. ***Number of Walk/Carries:*** ____

Date: ________________ **Page #** ____ **of** ____

Form #: LBW03222004
